# Time-varying MVAR algorithms for directed connectivity analysis: Critical comparison in simulations and benchmark EEG data

**DOI:** 10.1371/journal.pone.0198846

**Published:** 2018-06-11

**Authors:** Mattia F. Pagnotta, Gijs Plomp

**Affiliations:** Department of Psychology, University of Fribourg, Fribourg, Switzerland; Universidad Rey Juan Carlos, SPAIN

## Abstract

Human brain function depends on directed interactions between multiple areas that evolve in the subsecond range. Time-varying multivariate autoregressive (tvMVAR) modeling has been proposed as a way to help quantify directed functional connectivity strengths with high temporal resolution. While several tvMVAR approaches are currently available, there is a lack of unbiased systematic comparative analyses of their performance and of their sensitivity to parameter choices. Here, we critically compare four recursive tvMVAR algorithms and assess their performance while systematically varying adaptation coefficients, model order, and signal sampling rate. We also compared two ways of exploiting repeated observations: single-trial modeling followed by averaging, and multi-trial modeling where one tvMVAR model is fitted across all trials. Results from numerical simulations and from benchmark EEG recordings showed that: i) across a broad range of model orders all algorithms correctly reproduced patterns of interactions; ii) signal downsampling degraded connectivity estimation accuracy for most algorithms, although in some cases downsampling was shown to reduce variability in the estimates by lowering the number of parameters in the model; iii) single-trial modeling followed by averaging showed optimal performance with larger adaptation coefficients than previously suggested, and showed slower adaptation speeds than multi-trial modeling. Overall, our findings identify strengths and weaknesses of existing tvMVAR approaches and provide practical recommendations for their application to modeling dynamic directed interactions from electrophysiological signals.

## Introduction

All sensory and cognitive processes, including resting state activity, arise from the coordinated activity of multiple brain areas [1–5]. Brain areas continuously coordinate their activity through directed interactions, with activity in one area driving the activity in other areas through direct synaptic projections. To be useful, these inter-areal interactions must happen on small time scales, of the order of tens of milliseconds [6,7]. A better characterization of how directed network interactions evolve over time and under varying experimental conditions is crucial for understanding the functional role of single areas, as well for determining periods of network stability and change [8–12]. An important challenge, therefore, is how to derive estimates of directed connectivity between brain areas from multiple simultaneously recorded neurophysiological time series, as obtained with high temporal resolution using electroencephalography (EEG), magnetoencephalography (MEG) or local field potential (LFP) recordings.

Time-varying multivariate autoregressive (tvMVAR) modeling is a parametric approach to estimate dynamic interactions from physiological signals and derive measures of directed functional connectivity [13–16]. In this framework, algorithms based on recursive estimation were developed to provide valid models of non-stationary neural data [17–21]. Several such algorithms have been successfully used to characterize dynamic network interactions in sensory and motor processing [22–27], cognitive tasks [28], and pathological activity in epileptic patients [15,29,30].

Recursive algorithms for tvMVAR modeling require the a priori choice of two parameters: the model order and the adaptation coefficient. The model order is the maximum number of lagged observations included in the model. Several information criteria can be used to select an optimal model order [31–35], of which Akaike’s information criterion (AIC) and Bayesian information criterion (BIC) are most often used. Unfortunately, information criteria in practice often disagree about the optimal model order because they minimize different contributes or they may not converge to an optimal order at all [36]; these limitations strongly motivate an evaluation of the robustness of tvMVAR methods to variations in model order.

The adaptation coefficients are used in recursive algorithms to regulate the adaptation speed of parameters estimation and have to be selected between zero and one [17,18]. Values close to one lead to a faster adaptation (‘adaptivity’) but also a greater variance of parameter estimates, and this trade-off holds vice versa for values close to zero [37,38]. Thus, if the adaptation coefficients are not properly tuned, the performance of the recursive algorithm may be significantly degraded.

While tvMVAR algorithms have been previously tested in simulations [17,20,37,39], a systematic investigation into their robustness against parameter changes in real data is still missing. We therefore critically compared four algorithms that are commonly used to model non-stationary neurophysiological signals: the Recursive Least Squares (RLS) algorithm [18] and three algorithms based on Kalman filter, which are the General Linear Kalman Filter (GLKF) [17], the multivariate adaptive autoregressive (MVAAR) estimator [20], and the Dual Extended Kalman Filter (DEKF) [19,21].

When multi-trial time series are available, information from single trials can be combined in tvMVAR models to improve estimation accuracy and reliability of connectivity estimates [38]. Two strategies can be adopted to make use of multiple realizations: i) single-trial tvMVAR modeling followed by averaging across trials [40,41]; ii) multi-trial modeling, in which one tvMVAR model is simultaneously fitted to all trials [17,18,38]. The relative advantages of each approach and their sensitivities to parameter settings have not been systematically tested, but are important to understand when using these techniques in real data.

Here we provide a critical and comprehensive evaluation of the four recursive algorithms for tvMVAR modeling and the two ways of exploiting multiple realizations. To do so, we first used well-controlled simulated data and then exploited real benchmark EEG recordings that were previously obtained from rats in a somatosensory experiment where the ground truth is known [42,43]. In simulations and real data we measured both model quality and the accuracy of the estimated connectivity strengths and dynamics, while varying adaptation coefficients, model order, and sampling rate. We included variations in sampling rate because downsampling is commonly used in M/EEG and LFP analyses, but how this procedure affects the estimation accuracy of tvMVAR algorithms using these data is not well understood yet.

## Methods

### Time-varying MVAR models

The general form of a *d*-dimensional tvMVAR process of order *p* can be expressed as:

$$Y(n)=\sum_{r=1}^{p}A_{r}(n)Y(n-r)+E(n)$$ (1)

For each time step *n = 1*,*2*,…*N* the MVAR coefficients matrix *A*_*r*_*(n)∈R*^*dxd*^ and *E(n)* is a zero-mean uncorrelated *d*-dimensional white noise vector process.

We considered four recursive algorithms to estimate tvMVAR models. The first was the Recursive Least Squares (RLS) algorithm [18], which extends the Yule–Walker equations for the estimation of MVAR processes to the nonstationary case. For the adaptive estimation of the MVAR coefficients matrix RLS uses a forgetting factor *λ* that weights the error function stepwise in time and has to be selected a priori between 0 and 1. The algorithm initializes the update term *C* as a *dp*-by-*dp* matrix of zeros. Then the recursive estimation at each step is obtained by repeating the following computations for *n = p+1*,…,*N*:

$$\begin{array}{c} X_{n}=(x_{n-1},\ldots ,x_{n-p})\\ C_{n}=(1-\lambda )C_{n-1}+X_{n}^{T}X_{n}\\ \begin{array}{c} K_{n}=X_{n}C_{n}^{-1}\\ Z_{n}=x_{n}-X_{n}{A(n-1)}^{T}\\ A(n)=A(n-1)+Z_{n}^{T}K_{n} \end{array} \end{array}$$ (2)

with *X* being the observations on the previous *p* lags, *K* the gain matrix, *Z* the innovation matrix, and *A* the matrix of the MVAR coefficients of dimension *d*-by-*dp*. In this recursive estimation, the gain matrix gives more weight to measures with lower variance. The innovation is computed as the difference between observed and expected data and used to update the MVAR coefficients matrix.

The other algorithms here considered are based on the Kalman filter. The General Linear Kalman Filter (GLKF) algorithm [17,44] is one of them and is defined by two equations: an observation Eq (3), which connects the state process with the observation, and a state Eq (4), which models the state process as a random walk process.

$$O_{n}=H_{n}Q_{n}+W_{n}$$ (3)

$$Q_{n}=G_{n-1}Q_{n-1}+V_{n}$$ (4)

where the index *n* determines the time instant at which the estimation is performed, *O* indicates the observations, *H* is the transition matrix, *Q* is the state process, *W* is an additive observation noise, *G* is a transition matrix of a random walk process, and *V* is an additive process noise. The state process is defined in terms of parameter matrix, *A*_*r*_*(n)* from Eq (1), as follows:

$$Q_{n}=\left[\begin{array}{c} A_{1}{\left(n\right)}^{T}\\ \vdots \\ A_{p}{\left(n\right)}^{T} \end{array}\right]$$ (5)

When multiple trials are available, GLKF allows for single-trial modeling as well as multi-trial modeling. In the latter approach, the expected value of the additive observation noise covariance matrix is computed at each step with a recursive equation, in which the update term is obtained from the average covariance matrices of prediction error across *k* trials [17,45]:

$$W_{0}=I_{d},\;W_{n}=W_{n-1}(1-c_{1})+c_{1}{\left(O_{n}-Q_{n-1}\right)}^{T}(O_{n}-Q_{n-1})/(k-1)$$ (6)

where *I*_*d*_ is a *d*-dimensional identity matrix, and the other terms come from Eqs (3) and (4). The algorithm uses two adaptation constants *c*_*1*_ and *c*_*2*_ that play a role similar to the forgetting factor in RLS, and also have to be set between zero and one. The constants *c*_*1*_ regulates the proportion between estimates at the previous step and the update term in Eq (6), while the constant *c*_*2*_ weights the expected value of the additive process noise covariance matrix, which is estimated constantly as a weighted identity matrix of dimension *dp*-by-*dp*, as follows:

$$V_{n}=c_{2}I_{dp}$$ (7)

By tuning the two adaptation constants it is possible to regulate the speed of adaptation to transitions in temporal dynamics of connectivity patterns. High values increase adaptation speed but increase also estimation variance, while, low values smooth estimates in time by reducing variance but also speed in adaptation.

A second Kalman filter algorithm is the multivariate adaptive autoregressive (MVAAR) estimator [20]. In this algorithm the measurement noise covariance matrix is updated using the prediction error of the previous step [45], while estimating the covariance of the additive matrix noise of the state process using a variant proposed by Isaksson and colleagues [46]:

$$V_{n}=c_{2}^{2}I_{dp}$$ (8)

where *I*_*dp*_ is the identity matrix of dimension *dp*-by-*dp*.

A third variant of the Kalman filter, called Extended Kalman Filter, was developed to provide efficient maximum-likelihood estimates of discrete-time nonlinear dynamical systems [47]. In the Dual Extended Kalman Filter (DEKF) [19,21], which is tested here, both the states of the dynamical system and its parameters are estimated simultaneously. Similarly to GLKF and MVAAR, an update coefficient has to be set between zero and one to regulate how much estimates from the previous step are included for estimation at the current step. We here used the freely available implementation of DEKF (https://www.mathworks.com/matlabcentral/fileexchange/33850-dual-extended-kalman-filter--dekf-).

When multiple trials are available, RLS and GLKF allow for both single-trial and multi-trial modeling; while DEKF and MVAAR only allow for single-trial modeling, because the multi-trial approach is currently not implemented for them. In this study we thus critically evaluated the following algorithms: i) RLS using either single-trial modeling (RLS-ST) or multi-trial modeling (RLS-MT); ii) GLKF using either single-trial modeling (GLKF-ST) or multi-trial modeling (GLKF-MT); iii) DEKF using single-trial modeling (DEKF-ST); iv) MVAAR using single-trial modeling (MVAAR-ST).

The Partial Directed Coherence (PDC) [48] is a spectral MVAR-based connectivity measure, which is able to distinguish direct from indirect connections. To infer time-varying connectivity from the different tvMVAR models we used a squared variant of the PDC in which the information flow from *j* to *i* is normalized by the total amount of inflow to *i* [48,49]:

$${PDC}_{ij}(f,t)=\frac{{\left|A_{ij}(f,t)\right|}^{2}}{\sum_{m=1}^{d}{\left|A_{im}(f,t)\right|}^{2}}$$ (9)

This measure has been previously well-validated and tested [42,49–51]. Unless specified otherwise, time-frequency connectivity analyses were performed up to Nyquist frequency.

### Numerical simulations

We used two surrogate networks, one with 5 nodes and the other with 2 nodes, which were simulated as vector autoregressive processes with time-varying causal influences between nodes as in Eq (1). In order to simulate measurement noise we added uncorrelated white Gaussian noise to the time series of each node in each simulated condition. The variance of these noise terms was adjusted to produce a signal-to-noise ratio of 20 dB, which is here defined as the ratio of signal variance and noise variance.

**Fig 1:**
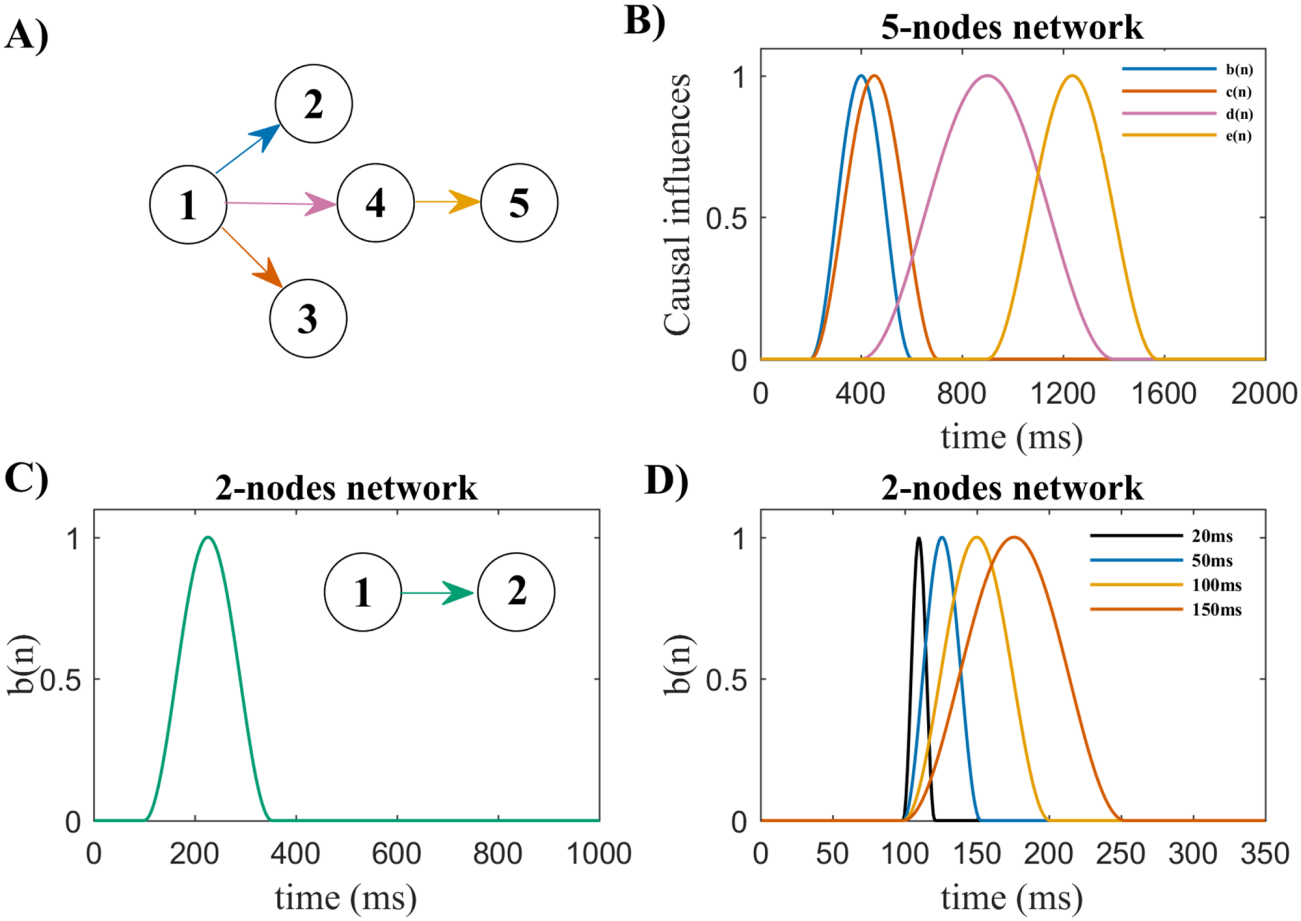
Simulated networks and time courses of the causal influences imposed. A) The diagram highlights the directed connections (arrows) imposed in the 5-nodes network, which was used for Simulation 1. All remaining possible connections between nodes are imposed to be constantly equal to zero. B) Shows the time-courses of the causal influences imposed in the 5-nodes network (Simulation 1), i.e. the dynamic evolution of the strength of each directed connection imposed in the model. Color coding matches the colors of the arrows shown in A). C) The diagram shows the directed connection imposed from node 1 to node 2 (green arrow) in the 2-nodes network and its time course in Simulation 2. This influence is active for a total duration in time of 250 ms. D) Alternative time courses of the causal influence from node 1 to node 2, with varying total durations, are considered for Simulation 3, which makes use of the 2-nodes network.

For the 5-nodes network the diagram in Fig 1A provides the layout of directed connections that are active at some point in time during each trial. All the other possible connections between nodes were constantly set to zero. Trials were simulated with a length of 2 seconds, considering 1000 time points at a sampling frequency of 500 Hz. The parameters *b(n)*, *c(n)*, *d(n)* and *e(n)* denote the time courses of causal influences imposed in the network (Fig 1B), i.e. each represents how the strength of the directed connection between a specific pair of nodes changes over time in every trial. For each imposed connection we imposed a lag in the autoregressive model. This lag represents the delay (in time samples) with which the signal of sender node enters in the prediction of the signal of receiver node. The imposed lags for *b(n)*, *c(n)*, *d(n)* and *e(n)* were 1 (2ms), 1 (2ms), 2 (4ms) and 3 (6ms) time points, respectively.

The simpler 2-nodes network was used to test how well each algorithm models causal influences of varying durations. In this network, the time course of the parameter *b(n)* denotes the intensity of the causal influences from node 1 towards node 2 (Fig 1C), and different values were considered for the model order. Varying durations of causal influences from node 1 to node 2 were considered (Fig 1D). In the second model, we used a sampling frequency of 1000 Hz and trial duration of 1 second to generate the process.

For both networks, we simulated datasets of 20 trials, and repeated the generation-estimation procedure 50 times for each of the conditions considered. We performed a total of three simulations.

By using the general term ‘adaptation coefficients’ we henceforth refer to adaptation constants in Kalman filter algorithms, forgetting factor in RLS, and update coefficient in DEKF. In Simulation 1, using the 5-nodes network (Fig 1A and 1B), we varied adaptation coefficients from 0.001 to 0.7 in 18 logarithmic steps. We considered a fixed model order *p* = 3 for tvMVAR fitting.

In Simulation 2, we used the 2-nodes network (Fig 1C) imposing different lags for the causal influence (4, 8, 12, 16 or 20 ms) and varied model orders (between 2 and 22 at step of 2).

In Simulation 3, we used the 2-nodes network (Fig 1C) and downsampled the generated time series (1000 Hz) using 10 sampling rate levels, from 1000 Hz to 100 Hz in steps of 100 Hz. A zero-phase antialiasing filter was used before downsampling to mitigate distortions due to aliasing. To take into account the fact that after downsampling the causal influence can be observed only through a decimated number of samples, we repeated the analysis using different durations of the imposed causal influence (Fig 1D). The causal influence from node 1 to node 2 (Fig 1C and 1D) had fixed lag of 10 ms, and model orders were chosen to match this lag at each sampling frequency. We here performed time-frequency connectivity analysis up to 50 Hz, which is the Nyquist frequency at lowest sampling rate.

We assessed performance of each algorithm in three ways: by quantifying tvMVAR model quality, by quantifying how well connectivity results reflected the simulated connectivity structure, and by assessing whether the timing of the dynamic interactions were correctly represented.

We used two measures of model quality: goodness-of-fit (GOF) and percent consistency. GOF reflects how much of the signal is explained by the model parameters [20], and is defined as [1-REV]*100, where REV is the relative error variance and is obtained as mean squared error (MSE) [52], i.e. the mean of the squares of the differences between observed values of the time series and values recreated from the MVAR coefficients, normalized by the variance of the observed signal. The percent consistency checks instead what proportion of the correlation structure in the data is accounted for by the model [14].

Since we simulated each network as a tvMVAR process, the simulated PDC values can be derived directly from the coefficients matrix used in each simulation itself, providing known time-frequency connectivity values for each edge in the network. We evaluated connectivity estimation accuracy by computing misses and false alarms as the normalized mean squared differences between the estimated PDC and the simulated PDC. Squared differences were calculated for each time-frequency point and then averaged across time points and frequencies, separately for edges with simulated connections (misses) and edges without simulated connections (false alarms). Both measures were successively normalized with respect to the mean squared simulated PDC values on edges with simulated connections. For example, if we consider the simple 2-nodes model (Fig 1C), misses were computed on the edge from 1 to 2 and false alarms were computed on the edge from 2 to 1. The closer the measures are to zero, the better the connectivity estimation accuracy.

Furthermore, we defined a measure of peak delay as the average difference between estimated peak latency and simulated peak latency, evaluated on edges with simulated connections. Values of peak delay close to zero indicate correct estimation of the timing of the imposed dynamic causal influence.

### Benchmark EEG data

In order to compare tvMVAR algorithms in real data we used previously recorded epicranial multichannel EEG from ten rats during unilateral whisker stimulations [43], where structural pathways are relatively well known and the physiology has been intensively investigated, providing strong expectations about a specific configuration of functional connections between cortical areas. For this reason, this dataset allows for direct comparisons between algorithms according to previously proposed performance criteria [42], detailed below.

**Fig 2:**
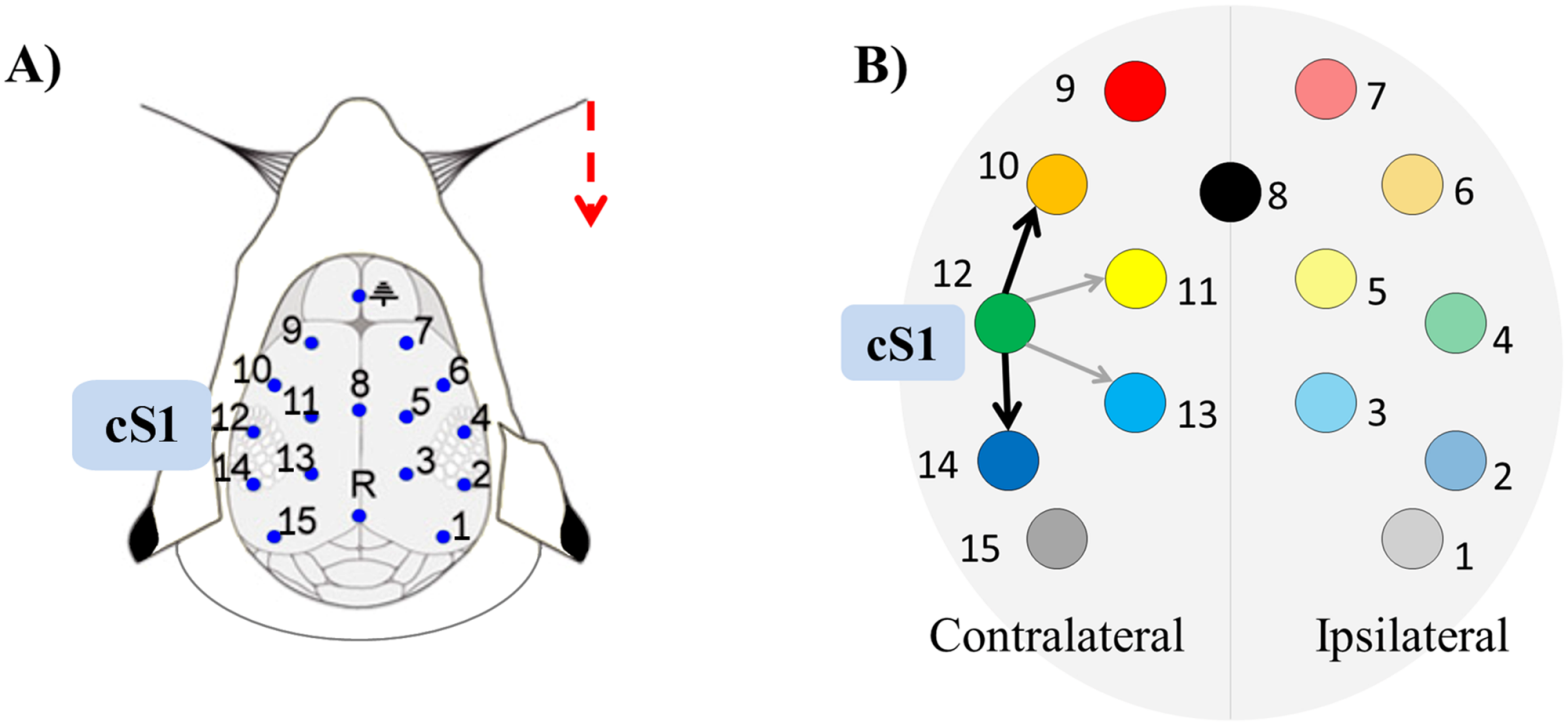
Benchmark EEG data: Recording setup and network of expected functional connections. A) Provides a schematic representation of the setup for epicranial recording. The dashed arrow in red represents the unilateral whisker stimulation. B) The diagram shows the expected behavior of the cortical network’s connections at early latencies after whisker stimulation. This is characterized by dominant total driving from contralateral primary somatosensory cortex (cS1, node 12) at latencies between 8 and 14 ms after stimulation. At peak driving the preferential directions of cS1 connections are expected to be found towards contralateral parietal (node 14) and more frontal cortex (node 10). Bright colors are used for nodes on the contralateral hemisphere to stimulation, while pale colors are used for the nodes on the ipsilateral hemisphere.

Animal handling procedures were approved by the Office Vétérinaire Cantonal (Geneva, Switzerland) in accordance with Swiss Federal Laws. In the recording setup, while the rat was under light isoflurane anesthesia, a multielectrode grid was placed in contact with the skull of the animal (Fig 2A). Signals were acquired using a sampling rate of 2000 Hz and bandpass filtered online between 1 and 500 Hz. A total of 15 channels were recorded and these provided the nodes of the network for our analyses (Fig 2B). These data are freely available (https://doi.org/10.6084/m9.figshare.5909122.v1) and further details about the recording procedure can be found elsewhere [42,43].

We used a semi-automatic procedure to remove trials contaminated with artifacts (see S1 Appendix); the average number of remaining trials per animal was 65 (range 34–80). After preprocessing, we estimated an optimal model order *p* = 8, which corresponds to a lag of 4 ms at original sampling rate (2000 Hz), by taking the median of the distribution across animals of optimal values from AIC (range 7–27) and BIC criteria (range 4–16). We opted for this approach to obtain one unique model order across animals that could be scaled across sampling rates and used in the two analyses where the model order was not explicitly varied.

As in the simulations, we assessed performance while varying adaptation coefficients, model orders and sampling rate. First, we evaluated the effect of varying adaptation coefficients, using the same range of values previously used in simulations. For this analysis we set the sampling rate to 500 Hz. This choice still guarantees good temporal resolution (2 ms) and sufficiently broad frequency range to correctly investigate whisker-evoked cortical interactions, while at the same time reducing computational time and model complexity, which can be particularly problematic for some recursive algorithm, as we will show later.

We then varied model order between 2 and 16 at step of 2, using fixed sampling rate (2000 Hz) and adaptation coefficients (0.02). Finally, to evaluate the effect of downsampling we used sampling rates of 2000, 1000 and 500 Hz, adjusting model order to match the 4 ms lag and keeping adaptation coefficients fixed. In each condition evaluated, the time-varying spectral connectivity matrices obtained with the different algorithms were averaged in the gamma-band (40–90 Hz), which is the predominant frequency over contralateral primary somatosensory cortex (cS1) [42].

We evaluated model quality using GOF and percent consistency, and systematically compared connectivity performance according to three previously proposed criteria [42], which are related to key characteristics expected in the functional network evoked by whisker stimulation (Fig 2B). Because whisker-evoked activity propagates from primary somatosensory cortex in the contralateral hemisphere (cS1; node 12 in Fig 2B), strong functional outflow is expected from cS1 at early latencies. The functional connections from cS1 are expected to preferentially target frontal sensory-motor and parietal regions (nodes 10 and 14 in Fig 2B), because of strong structural connectivity from cS1 with these regions and in line with the sequential activation pattern observed in somatosensory evoked potentials [42,43]. Thus, the three previously proposed performance criteria were defined as ability to: detect cS1 as the main driver of the network (criterion I); identify peak-driving from cS1 at physiologically plausible latencies, between 8 and 14 ms after stimulus onset (criterion II); correctly distinguish the main targets of cS1, i.e. contralateral parietal and frontal cortex (criterion III) [42].

The total driving from a region was defined as the sum of all outflows (PDC) from that region. In order to evaluate how well cS1 (the expected dominant driver) could be distinguished (criterion I), the total driving from cS1 was statistically compared to the total driving from the second largest driver, at the latency of peak-driving from cS1. We evaluated whether the observed difference between the two drivers reliably exceeded zero by using a bootstrap approach. We first computed the difference between cS1’s total driving and that of the second largest driver for each rat. We then resampled the observed differences with replacement to create a bootstrap distribution of differences (n = 10,000), where the size of each resample is equal to the size of the original dataset [53]. For constructing 95% confidence intervals (CIs) from the bootstrap distribution we used the bias-corrected and accelerated (BCa) method, which corrects for bias and skewness in the distribution of bootstrap estimates [54,55]. If lower 95% CI of bootstrapped pairwise differences exceeded zero, this was taken to indicate a significant difference between cS1 and the second largest driver, and consequently a reliable identification of cS1 across rats. The fact that this comparison was done at peak latencies cS1 poses no problem of circularity because the comparison is against the second largest driver, not against the null hypothesis of no driving.

To evaluate the main targets of cS1 (criterion III), the connections from cS1 toward contralateral parietal (node 14, Fig 2B) and frontal sensory-motor regions (node 10, Fig 2B), which in Fig 2B are denoted by black arrows, were statistically compared with those toward the corresponding medial electrodes equidistant from cS1, nodes 13 and 11 (grey arrows in Fig 2B), respectively, with null hypothesis of no difference between directions, and by using the same nonparametric bootstrapping approach described for criterion I. In each comparison we then calculated effect size using Cohen’s *d* with pooled standard deviation in the denominator [56].

## Results

### Simulation results

#### Simulation 1: Adaptation coefficients

**Fig 3:**
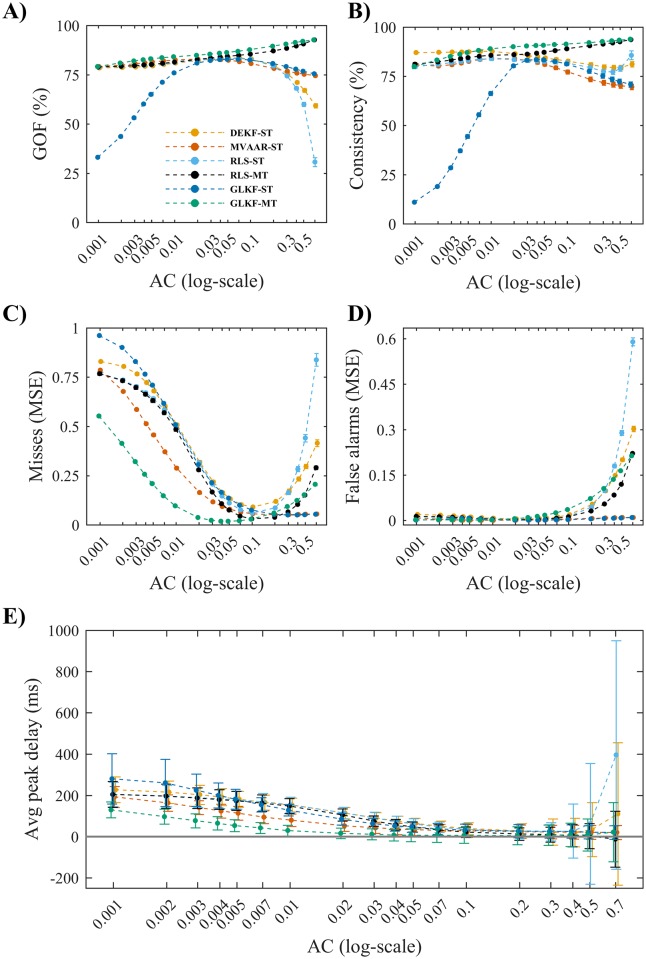
Simulation 1 on the effects of varying adaptation coefficients. A) Shows the goodness-of-fit (GOF). B) Shows the percent consistency. C) Shows the misses (normalized MSE), which are computed on the edges with simulated connections (Fig 1A). D) Shows the false alarms (normalized MSE), computed on the edges with null connections. E) Shows the average peak delay across the four imposed connections in the 5-nodes model (Fig 1B). The results in each plot are shown as a function of adaptation coefficients AC, using logarithmic scale for the x-axis, for the four recursive algorithms and the two ways of exploiting multiple trials: single-trial modeling (ST) and multi-trial modeling (MT), available only for RLS and GLKF. Error bars represent 95% CI of the mean value computed across 50 simulations.

In Simulation 1 we evaluated the effects of varying adaptation coefficients. The results showed good model fits with monotonic increases of GOF (Fig 3A) and consistency (Fig 3B) for increasing adaptation coefficients for GLKF-MT and RLS-MT. For the remaining algorithms, we observed degradations in model fitting for adaptation coefficients above 0.05. In particular, GLKF-ST showed the strongest dependence on adaptation coefficients, resulting in poor model fit also for small adaptation coefficients.

Differences in model quality did not directly translate to differences in connectivity performance (Fig 3C and 3D). All algorithms showed accurate estimation of the simulated connections and small MSE in false alarms for values of adaptation constants close to 0.1. For even higher adaptation coefficients, MVAAR-ST and GLKF-ST showed better accuracy in connectivity estimation, while the remaining four algorithms showed increased misses and false alarms. All six algorithms showed poor estimation of simulated connections (misses) with small adaptation coefficients, due to inability in rapidly following the temporal dynamics of the causal influences in the simulated process.

Increasing adaptation coefficients also improved the ability of all algorithms to correctly detect peak latencies of driving, resulting in average peak delay close to zero already with adaptation coefficients of 0.02 for GLKF-MT, and above 0.2 for the others (Fig 3E). Overall, values of peak delay closer to zero were obtained with GLKF-MT. When using adaptation coefficients above 0.2, however, the results showed higher variability in peak latencies detection across datasets for GLKF-MT. In the same range above 0.2, high variability in results was found also for the two RLS algorithms and DEKF-ST.

In sum, Simulation 1 showed how adaptation coefficients can strongly affect connectivity results and that coefficients optimal for one algorithm may not be optimal for another algorithm under the same experimental conditions. In general, the optimal adaptation coefficients further depend on the dynamics of the investigated phenomena.

#### Simulation 2: Model order

**Fig 4:**
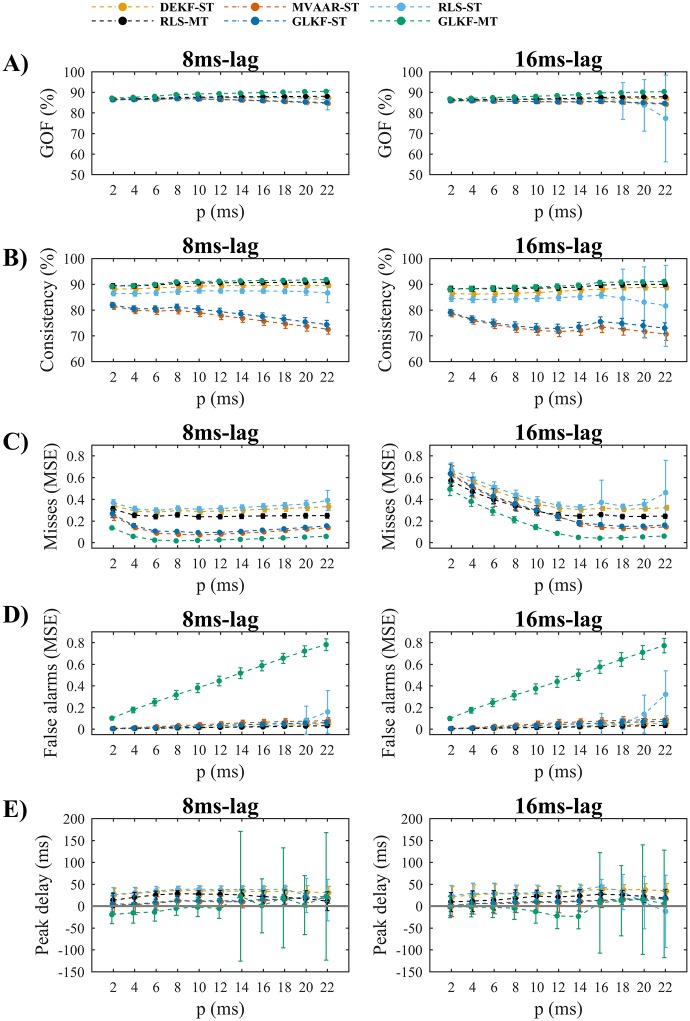
Simulation 2 on the effects of model order selection. A) Shows the goodness-of-fit (GOF). B) Shows the percent consistency. C) Shows the misses, which are computed as normalized mean squared differences between estimated and simulated values of the imposed causal influence from node 1 to node 2 (Fig 1C). D) Shows the false alarms, which are computed as normalized mean squared error on the null connection from node 2 to node 1 (Fig 1C). E) Shows the peak delay, which is computed as difference between peak latencies of estimated and simulated causal influence from node 1 to node 2 (Fig 1C). The results are shown for lags of the imposed causal influence of 8ms (left plots) and 16ms (right plots). In each plot the results are reported varying model order *p*, for the four recursive algorithms and the two ways of exploiting multiple trials: single-trial modeling and multi-trial modeling, available only for RLS and GLKF. Error bars represent 95% CI of the mean value computed across 50 simulations.

In Simulation 2 we tested the robustness against variations in model order with different lags of the imposed causal influence. Since we found similar results across lags, only representative results for 8 and 16 ms lags are shown in Fig 4.

All algorithms showed good (above 80%) and generally uniform GOF across model orders (Fig 4A). A similar behavior was observed in the percent consistency (Fig 4B) for most of the algorithms, except MVAAR-ST and GLKF-ST, which showed overall lower values of consistency, suggesting that signals re-created from these models more poorly reflect the original time series.

All algorithms showed similar behaviors in terms of misses (Fig 4C), characterized by minimum MSE for a model order close to the value matching the imposed lag. In general, we observed higher MSE for model orders smaller than the imposed lag and relatively stable MSE for a range of model orders that exceed that value. We found small values of MSE in false alarms that modestly increased with model order (Fig 4D) for all algorithms except GLKF-MT, which showed larger overestimations of absent connections that rapidly increased with model order.

Most algorithms systematically overestimated peak latencies across model orders (Fig 4E). Most accurate peak latency detection was obtained using the two GLKF algorithms and MVAAR-ST, which showed 95% CIs of peak delay that overlap with the line of zero delay for most model orders. Despite very good performance for small model orders, GLKF-MT showed large variability in the results across simulations with model orders above 12 and 14 for imposed lag of 8 and 16 ms, respectively.

In sum, Simulation 2 showed that increasing model order beyond the optimal value may lead to poorer model quality, although it does not strongly impair connectivity estimation, at least for the ranges tested here. An exception to this robustness against too large model orders is represented by GLKF-MT, which showed relatively large false alarms (MSE) and unstable estimates of peak latency for large model orders.

We used AIC and BIC to check the model orders suggested by these information criteria in the simulated conditions. BIC suggested model orders in the range 8–10 (across simulations) when the imposed lag was 8 ms and in the range 17–19 when the lag was 16 ms; as previously shown, both ranges stills guarantee good connectivity estimation (Fig 4). AIC, however, suggested higher model orders and often resulted in lack of converge in the range of values considered. For example, AIC resulted in model orders ranging between 22 and 40 (maximum tested) for the 16 ms lag. Differences in results can be explained by the fact that the BIC favors sparse solutions and penalizes the number of parameters more strongly than AIC. While the non-convergence problem was one of the motivations of our analysis, it does not seem to be strongly problematic because a broad range of model orders essentially result in very similar estimated connections.

#### Simulation 3: Downsampling

**Fig 5:**
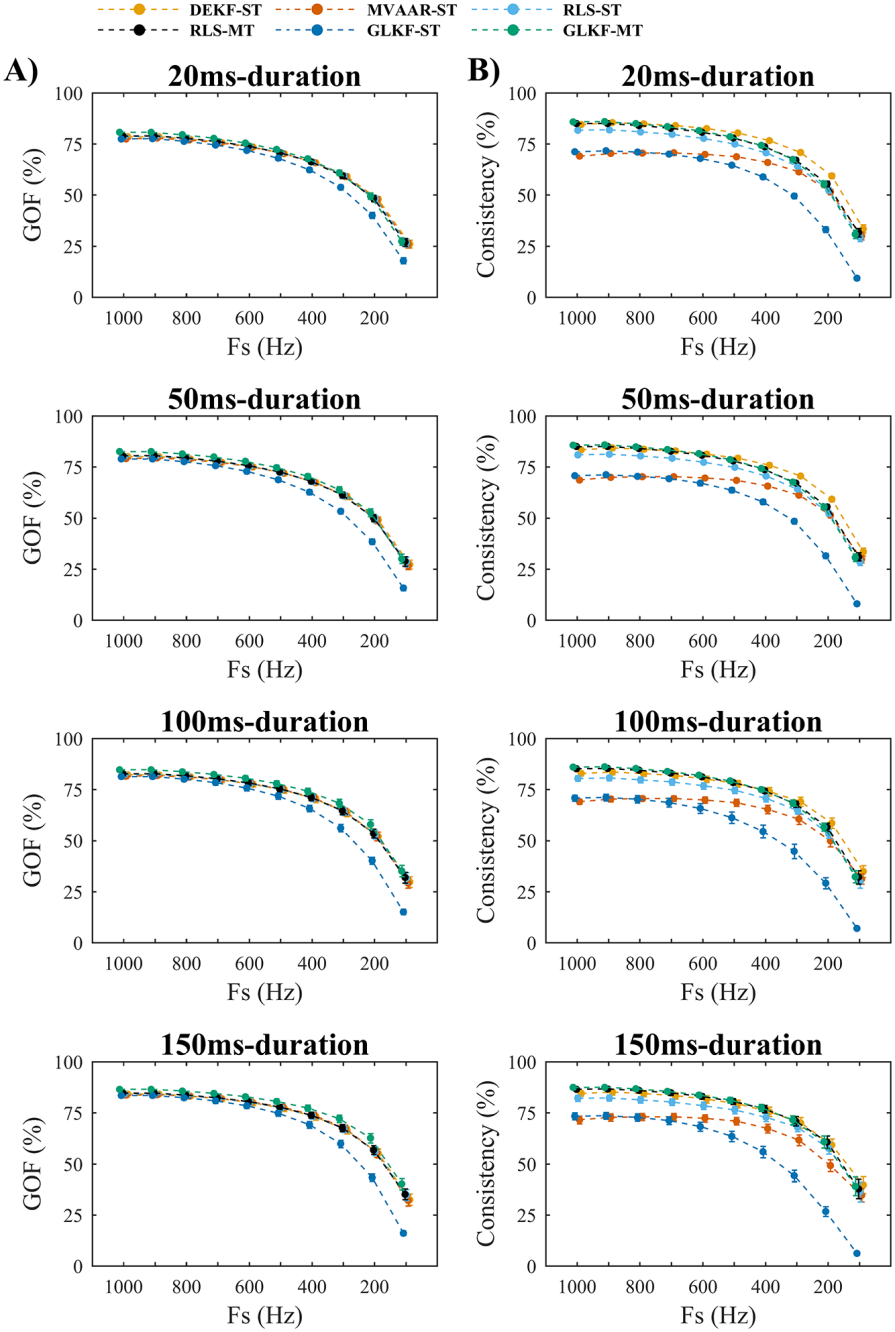
Simulation 3 on the effects of varying sampling rate: Model quality. A) Show the goodness-of-fit (GOF). B) Shows the percent consistency. The results are shown for four different durations of the imposed causal influence from node 1 to node 2 (Fig 1D): 20 ms (first row), 50 ms (second row), 100 ms (third row), and 150 ms (fourth row). In each plot the results are reported varying sampling rate Fs, for the four recursive algorithms and the two ways of exploiting multiple trials: single-trial modeling and multi-trial modeling, available only for RLS and GLKF. Error bars represent 95% CI of the mean value computed across 50 simulations.

**Fig 6:**
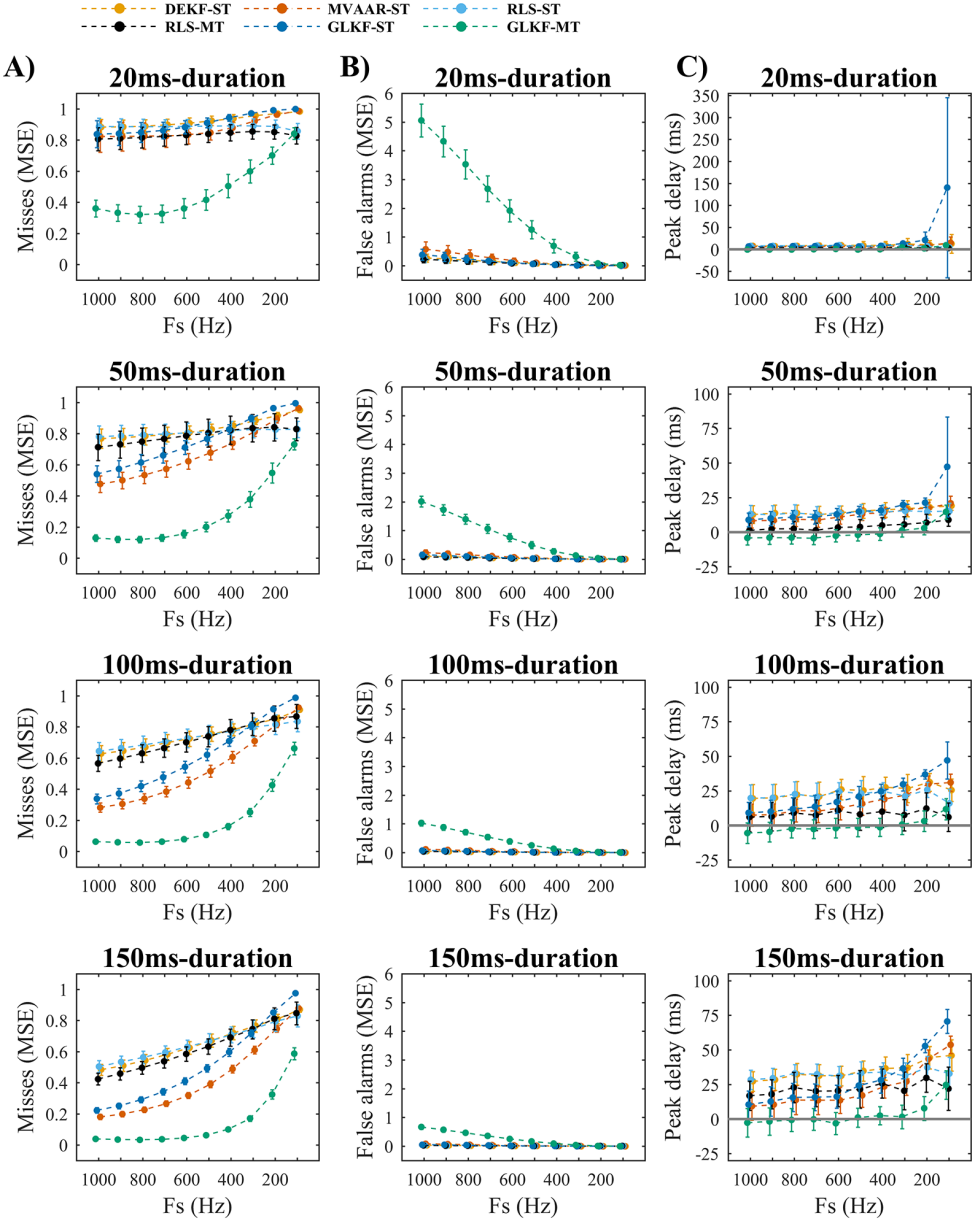
Simulation 3 on the effects of varying sampling rate: Connectivity estimation. A) Shows the misses (normalized MSE), which are computed on the edge from node 1 to node 2 (Fig 1C). B) Shows the false alarms (normalized MSE), which are computed on the edge from node 2 to node 1 (Fig 1C). C) Shows the peak delay, which is computed as difference between peak latencies of estimated and simulated causal influence from node 1 to node 2 (Fig 1C). The results are shown for four different durations of the imposed causal influence from node 1 to node 2 (Fig 1D): 20 ms (first row), 50 ms (second row), 100 ms (third row), and 150 ms (fourth row). In each plot the results are reported varying sampling rate Fs, for the four recursive algorithms and the two ways of exploiting multiple trials: single-trial modeling and multi-trial modeling, available only for RLS and GLKF. Error bars represent 95% CI of the mean value computed across 50 simulations.

Simulation 3 evaluated the effect downsampling on model quality (Fig 5) and connectivity estimation (Fig 6), using the 2-nodes network and varying durations of imposed causal influence (Fig 1C and 1D). All algorithms showed a monotonic decrease in model quality with downsampling (Fig 5), for all causal influence durations, reflecting the reduction of data points available for modeling. As in Simulation 1, MVAAR-ST and GLKF-ST showed overall lower consistency values (Fig 5B).

For connectivity estimation (Fig 6), downsampling affected the algorithms differently. GLKF-MT showed small and stable MSE for misses (Fig 6A) with sampling rates from 1000 Hz down to 600–400 Hz. Differently, for the other algorithms the minimum MSE in misses was obtained at the original high sampling rate (1000 Hz), while downsampling resulted in a monotonic increase in MSE. On the contrary, values of MSE in false alarms (Fig 6B) decreased with downsampling for all six algorithms, confirming that downsampling can reduce false-positives, and we observed the highest gradient of this reduction in GLKF-MT. Connectivity estimation depended on the duration of the imposed causal influence. With the shortest duration (20 ms), most algorithms showed very high values of MSE in misses (Fig 6A), even at original sampling rate, while for longer duration performance improved. This reflects the fact that estimation precision increases when more samples are available to model the dynamic evolution of the imposed influence, being adaptation coefficients the same.

In terms of temporal discrimination, while peak delays were generally constant over sampling rates (Fig 6C), increased delays were observed for most algorithms at the lowest sampling rates, particularly for the longer imposed causal durations (100 and 150 ms).

To summarize, lower sampling rates led to decreased model quality and connectivity estimation accuracy. The notable exception to this was that with lower sampling rates the false alarms (MSE) of GLKF-MT dropped substantially while misses (MSE) remained stable, resulting in a global improvement in connectivity estimation accuracy for reduced sampling rates.

### Benchmark EEG results

The results from simulations indicated that there is an optimal adaptation constant value for connectivity estimation, that most algorithms are relatively robust against setting the model order too high, and that downsampling is generally detrimental to connectivity estimation. While simulations provide a well-controlled test environment, real data are more complex in ways that could both help and hinder model quality and connectivity estimation. We therefore systematically compared the performance of tvMVAR algorithms using real benchmark EEG data along fixed criteria, varying adaptation constants, model order, and sampling rate.

#### Adaptation coefficients

**Fig 7:**
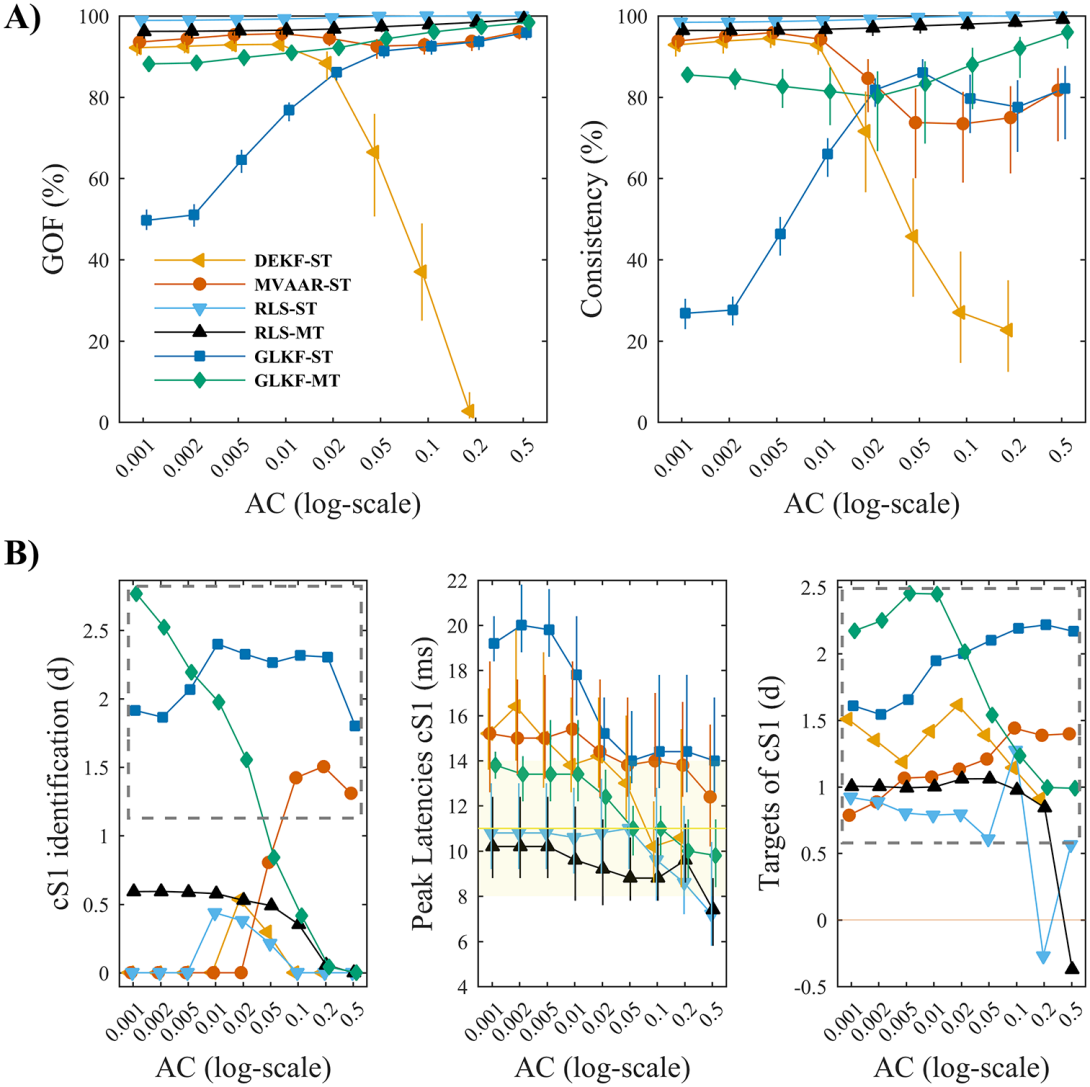
Effects of varying adaptation coefficients in benchmark EEG data. A) Shows the measures of model quality, goodness-of-fit (left) and percent consistency (right). Error bars denote bootstrapped 95% CI around the mean across animals. B) Shows the results of the performance assessment using the three performance criteria. Criterion I (left): gray dashed rectangle indicates conditions in which cS1 was significantly distinguished as main driver of the network. Criterion II (center): error bars denote bootstrapped 95% CI around mean peak latency across animals, while yellow band shows the range of physiologically plausible latencies after stimulus onset. Criterion III (right): gray dashed rectangle indicates conditions in which the driving from cS1 toward its two main targets was significantly larger than to medial electrodes equidistant from cS1. In each plot the results are shown as a function of the adaptation coefficients AC, using logarithmic scale for the x-axis, for the four recursive algorithms and the two ways of exploiting multiple trials: single-trial modeling and multi-trial modeling, available only for RLS and GLKF.

Most algorithms showed high GOF and consistency across values of adaptation coefficients (Fig 7A). Results on model quality reproduced our findings of Simulation 1 (Fig 3A and 3B) for most of the algorithms. We observed again that varying adaptation coefficients had little effect on model quality for algorithms based on multi-trial modeling, GLKF-MT and RLS-MT. For the other algorithms, differently from Simulation 1, we found increasing GOF with larger adaptation coefficients. We confirmed poor model fit with small adaptation coefficients for GLKF-ST and reduction in percent consistency with big adaptation coefficients also for MVAAR and DEKF.

As in Simulation 1, model quality did not directly translate to performance in connectivity inference (Fig 7B). For GLKF-MT, performance on criteria I (cS1 identification) and III (cS1 target identification) depended strongly on adaptation constant choice. Only adaptation coefficients in the range between 0.02 and 0.05 guaranteed good identification of the main driver of the cortical network and correct discrimination of its two targets, with big values of effect size (Cohen’s *d*), and also obtaining physiologically plausible latencies of the peak-driving from cS1 (criterion II). MVAAR-ST benefited from higher adaptation coefficients, as in simulations (Fig 3C–3E); while GLKF-ST appeared robust against variations of adaptation constants on criteria I and III. We also confirmed an intrinsic less accurate temporal discrimination for these last two algorithms, with values of peak latency (criterion II) outside the physiologically plausible range for adaptation coefficients below 0.05. Generally poor identification of cS1 as the main driver was observed for DEKF-ST and for RLS, irrespective of modeling approach.

#### Model order

**Fig 8:**
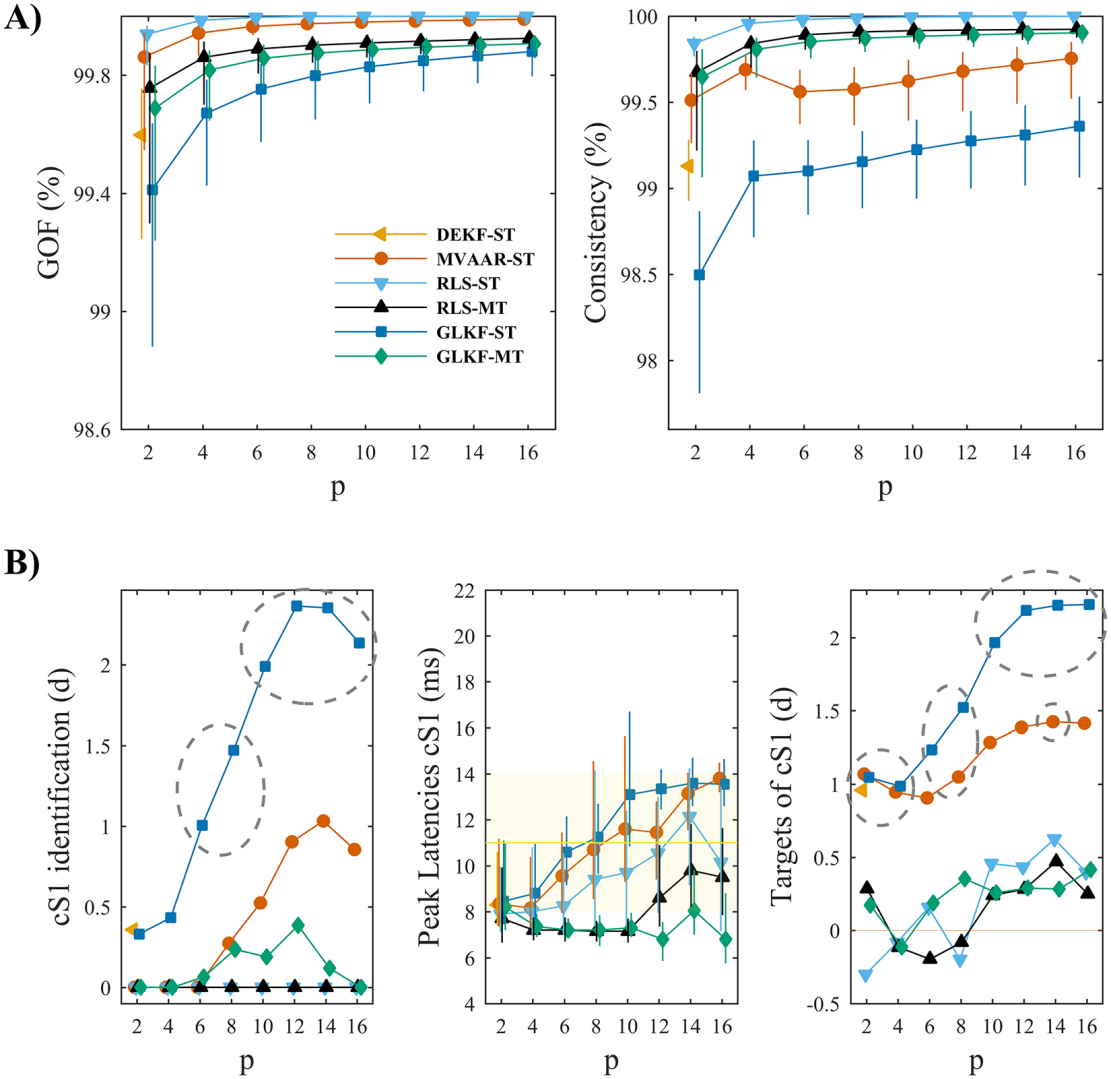
Effects of model order selection in benchmark EEG data. A) Shows the measures of model quality, goodness-of-fit (left) and percent consistency (right). Error bars denote bootstrapped 95% CI around the mean across animals. B) Shows the results of the performance assessment using the three performance criteria. Criterion I (left): gray dashed ellipses indicate conditions in which cS1 was significantly distinguished as main driver of the network. Criterion II (center): error bars denote bootstrapped 95% CI around mean peak latency across animals, while yellow band shows the range of physiologically plausible latencies after stimulus onset. Criterion III (right): dashed ellipses indicate conditions in which the driving from cS1 toward its two main targets was significantly larger than to medial electrodes equidistant from cS1. In each plot the results are shown varying model order *p*, for the four recursive algorithms and the two ways of exploiting multiple trials: single-trial modeling and multi-trial modeling, available only for RLS and GLKF.

Both model quality (Fig 8A) and connectivity performance (Fig 8B) in benchmark data generally improved with increasing model order. Improved GOF and percent consistency with increasing model order was found for all algorithms except DEKF-ST (Fig 8A).

In terms of connectivity inference (Fig 8B), only GLKF-ST showed plausible results, characterized by general improvement in the identification of cS1 (criterion I) and its targets (criterion III) when increasing model order, with stable performance for a broad range of values; for GLKF-ST most accurate peak latency identification (criterion II) was however obtained with intermediate model orders (around 8, the optimal model order as estimated from information criteria, see Methods). Furthermore, even if discrimination performance remained good, we found a slight reduction in *d* by increasing model from 14 to 16. Despite generally poor performance obtained with MVAAR-ST and GLKF-MT these algorithms showed a behavior in cS1 identification similar to GLKF-ST. Together these results suggest that a further increasing model order may at some point degrade connectivity inference, as already observed on criterion II for GLKF-ST and MVAAR-ST. Overall we found poor performance for DEKF-ST and the two RLS algorithms.

#### Downsampling

**Fig 9:**
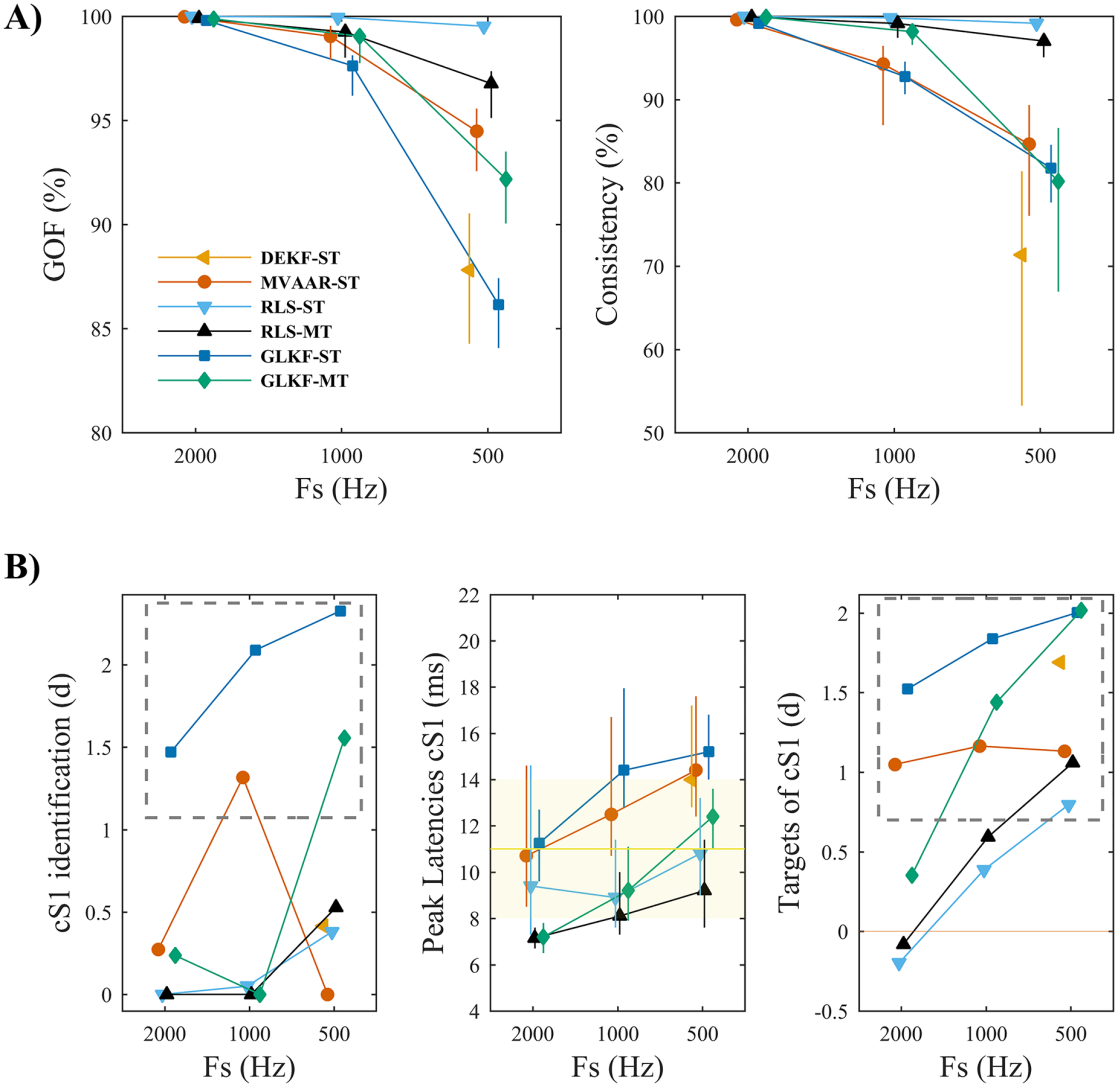
Effects of varying sampling rate in benchmark EEG data. A) Shows the measures of model quality, goodness-of-fit (left) and percent consistency (right). Error bars denote bootstrapped 95% CI around the mean across animals. B) Shows the results of the performance assessment using the three performance criteria. Criterion I (left): gray dashed rectangle indicates conditions in which cS1 was significantly distinguished as main driver of the network. Criterion II (center): error bars denote bootstrapped 95% CI around mean peak latency across animals, while yellow band shows the range of physiologically plausible latencies after stimulus onset. Criterion III (right): gray dashed rectangle indicates conditions in which the driving from cS1 toward its two main targets was significantly larger than to medial electrodes equidistant from cS1. In each plot the results are shown varying sampling rate Fs, for the four recursive algorithms and the two ways of exploiting multiple trials: single-trial modeling and multi-trial modeling, available only for RLS and GLKF.

While most algorithms showed GOF and percent consistency values close to 100% (Fig 9A), downsampling reduced model quality to varying degrees. Most robust to downsampling was RLS-ST while the other algorithms showed reduced, though still good, model quality with downsampling. These results resemble those obtained from Simulation 3 (Fig 5).

Consistently good connectivity performance across sampling rates was obtained only with GLKF-ST (Fig 9B). In fact, at the original sampling rate of 2000 Hz only GLKF-ST reliably identified cS1 as the largest driver, with a large effect size *d* = 1.47. After downsampling to 1000 Hz, also MVAAR-ST was able to significantly detect cS1; while, after downsampling to 500 Hz, only the two GLKF algorithms succeeded in distinguishing cS1 as the principal network driver, with effect sizes *d* = 2.33 for GLKF-ST and *d* = 1.56 for GLKF-MT.

On criterion II (Fig 9B), all algorithms identified peak driving from cS1 after stimulation at latencies close to the physiological range. Overall, we confirmed that the algorithms based on single-trial modeling have slower adaptation speed compared to those based on multi-trial modeling.

On criterion III (Fig 9B) performance tended to increase with downsampling. MVAAR-ST and GLKF-ST correctly identified the main targets of cS1 driving, across sampling rates; while GLKF-MT reliably identified cS1 targets only after downsampling. The remaining algorithms correctly identified both main targets of cS1 only after reducing sampling rate to 500 Hz. It is worth to notice that all the considered algorithms identified the contralateral parietal region as main target of cS1 in each dataset.

To summarize, our results in benchmark data showed that MVAAR-ST and GLKF-ST benefit from selecting adaptation coefficients higher (above 0.05) compared to the other algorithms, that a broad range of model orders result in basically the same estimated pattern of connections for all algorithms, and that downsampling significantly improved the performance of GLKF-MT.

## Discussion

We here systematically compared the performance of four recursive algorithms for tvMVAR model fitting and two strategies to deal with multiple realizations of the same stochastic process. To the best of our knowledge, this is the first study that exploits a combination of numerical simulations and real benchmark EEG data to provide such comparison and assess the effects of model parameters’ variation. In the first part of this work, we used numerical simulations to evaluate the intrinsic effects of model parameters on the final connectivity estimation under well-controlled conditions. In the second part of our study, we used real benchmark EEG data and varied the same model parameters; this permitted to assess the physiological plausibility of results obtained with these methods in more complex and highly dynamic signals.

Our findings show that among the algorithms tested GLKF [17] generally performs best in estimating dynamic causal influences between observed time series. This algorithm outperformed RLS and DLKF both in simulations and benchmark data, but its performance was shown to strongly depend on whether the single-trial (GLKF-ST) or multi-trial (GLKF-MT) approach is used. While the best performance can be obtained with GLKF-MT under specific circumstances (see below), the results suggest that in practice GLKF-ST may be more reliable, especially when using relatively high sampling rates and adaptation coefficients in the range between 0.02 and 0.05, because it is less prone to effects of overfitting than GLKF-MT. A drawback of GLKF-ST is that it has somewhat slower adaptation speed than GLKF-MT and this aspect may lead to a worse temporal discrimination of the causal influence.

The observed advantage of GLKF-MT in temporal discrimination comes from how it exploits multiple realizations (trials). With GLKF-MT the information from multiple realizations is combined and allows steeper variations in the recursive estimation when there is a dynamic change in the causal influence. As a consequence, GLKF-MT is generally characterized by faster adaptation and better temporal discrimination than GLKF-ST, but with higher variability in the estimates (as illustrated in S2 Appendix). Here GLKF-MT appeared in fact to be more sensitive to the problem of over-parametrization, especially when the model order was set too high for the process’ dynamics, or when the imposed lag of the causal influence was long with respect to the sampling interval. For these reasons, GLKF-MT must be recommended only when the time scales of the dynamic interactions are known and consequently also the proper decimation factor for downsampling, which may not always be the case in real data. When using high sampling rates, one could still use GLKF-MT but lowering adaptation coefficients (in the order of 0.001), so that both adaptation speed and variance in parameters estimates are reduced, obtaining performance characteristics similar to those of GLKF-ST with adaptation constants one order of magnitude higher, i.e. in the range 0.02–0.05.

With respect to GLKF-MT, GLKF-ST allows obtaining smoother estimates with smaller variance, and this reduces spurious estimates (false alarms); however, the intrinsic adaption speed of the algorithm remains the same as at the level of each single trial (see S2 Appendix for a simulation illustrating this point). Altogether these aspects explain why we found different performance for GLKF-ST and GLKF-MT, both in simulations and in benchmark data, while similar results were obtained across the two RLS algorithms, in line with previous findings showing that the adaptation speed in RLS is not affected by the number of trials, but only by the adaptation coefficient [57].

The results showed that performance similar to GLKF-ST can be obtained using MVAAR [58]. This similarity is a consequence of the fact that the variants adopted in MVAAR for the estimation of measurement noise covariance matrix and covariance of the additive matrix noise of the state process [45,46] are analogous to those implemented in GLKF. However, our results in real data show overall poorer performances and less robustness to variations in sampling frequency for MVAAR as compared to GLKF-ST, especially for the identification of major network drivers.

Based on the results of our study, we discourage the use of RLS [18]. It was already well known that RLS presents limitations in the analysis of high-dimensional time series, due to possible computational instabilities [17,39,51]. These instabilities explain the often uninterpretable results obtained using RLS algorithms in benchmark data, where we analyzed a 15-dimensional network. We obtained poor performance also using DEKF with single-trial modeling [21,59]. More specifically, DEKF showed significant computational instabilities in benchmark data, resulting in uninterpretable estimates, especially for high sampling rates and increased model orders (for further details see S3 Appendix). The DEKF algorithm has been successfully used for small networks in numerical simulations and real data [21,41,59,60]. Our results suggest, however, that an increase in number of nodes may be problematic for this algorithm. The computational limitations of the DEKF and RLS algorithms mean that unreliable results may arise from their application to high-dimensional networks.

In this study, we investigated the effects of varying the adaptation coefficients, which offer the possibility to tune the adaptivity of recursive algorithms, by choosing a trade-off between speed of adaptation and smoothness of the estimates [17,18,20,57]. We found that these coefficients can strongly affect connectivity results and that the optimal range of values for selecting them not only depends on connections’ dynamics, but also on the type of algorithm and its intrinsic characteristics. Our results showed dependence of model quality measures on the choice of adaptation coefficients, suggesting optimal values to maximize model fit to the observed time series. In principle, when analyzing real data, one could then optimize GOF for a certain range of adaptation coefficients and select values accordingly. However, we observed a discrepancy between model quality and connectivity estimation accuracy, both in simulations and benchmark data: models with the best fit to the data did not always have the best connectivity performance. As a consequence, optimizing adaptation coefficients purely on the basis of model quality does not necessarily minimize connectivity estimation errors. As a priori choice, when using GLKF-MT our results suggest adaptation coefficients in the range between 0.01 and 0.05, in line with previous studies [17,37,51]. The same range of values can be used also for the DEKF and RLS algorithms. Another study showed that, depending on the experimental conditions, the optimal value for the forgetting factor in RLS varies between 0.01 and 0.04 [61], as also suggested in [18]. In addition, increasing the amount of trials can reduce the variance of parameters estimation, up to the point where we can increase forgetting factor and consequently adaptation speed without losing accuracy in the estimation [57,61]. In terms of adaptation coefficients, we extended the findings of previous literature by showing that, under same conditions, MVAAR-ST and GLKF-ST benefit from selecting adaptation coefficients above 0.05, i.e. above the range optimal for the other algorithms. Our findings also suggest that the optimal range of values for the adaptation coefficients further depends on the sampling rate. In fact, with respect to our recommendations and those of previous studies, an exception occurs when high sampling rates are considered. In these cases a safer choice is to use values an order of magnitude lower, i.e. around 0.001 for GLKF-MT and in the range between 0.02 and 0.05 for GLKF-ST and MVAAR-ST.

In general, as in the case of the classical event-related potentials analysis, the amount of available trials can influence the estimation performance of recursive algorithms. An increase in number of trials has been shown to reduce estimates’ variance in RLS [57,61]. The influence of number of trials has also been evaluated for GLKF-MT in previous studies [39,51]. Using a simulation framework as in Simulation 1, separately we assessed the effects of amount of trials’ variation on the estimation performance of the six algorithms here considered (see S4 Appendix). Our findings confirm that increasing the amount of trials is beneficial for all recursive algorithms both in terms of connectivity estimation and temporal discrimination, but these positive effects become almost negligible rather soon. We show that connectivity performances are characterized by a plateau beyond a certain number of trials. In our simulation a number of trials above 20 guaranteed reliable connectivity estimation. However, in practice it is very difficult to establish a priori an exact number of trials needed for the estimation, because this critically depends on the quality of the recorded data, the network dimensionality, and the number of parameters to estimate.

Another important step in parametric autoregressive approaches is the selection of model order [36]. On the one hand, model order has to be selected sufficiently high to correctly describe the dependencies of a multivariate process and capture essential dynamics of the data; on the other hand, increasing model order may lead to overfitting effects with consequent increase in the variability of the successive MVAR model estimates [62]. Therefore, an inappropriate choice of model order may significantly degrade accuracy of the final connectivity estimates. Results in simulations showed that all algorithms were relatively robust to variations in model order. In fact, although the model order had to be selected sufficiently high to capture the delay of the imposed causal influence, we observed little changes in connectivity performance from increasing model order beyond the optimal value, for a reasonable range of values. Moreover, our results in benchmark data showed that a broad range of model orders result in basically the same estimated pattern of connections, confirming findings of previous studies [37,63]; in addition, neither model fitting criteria nor connectivity performance criteria showed clear maximal values for the optimal model order as derived from information criteria. Together our results suggest that when objective information criteria do not converge to an optimal model order, then the model order is better selected a bit too large than too small, while carefully taking into consideration any a priori knowledge about the timing of the observed phenomena.

Interestingly, we found that downsampling can have both beneficial and detrimental effects on connectivity estimation, depending on the algorithm used. Our results in simulations and benchmark data showed positive effects of downsampling on the performance of GLKF-MT, due to a reduction in estimates’ variability; while, in general downsampling degraded performance for the remaining algorithms. Previous studies investigated downsampling effects on Granger causality in fMRI recordings [64,65], showing that severe downsampling results in a failure of causality analysis. Downsampling may reduce the variability of causal estimates, by reducing model parameters to estimate [66,67]; however, after downsampling the causal influences can be observed only through a decimated number of samples, and this may significantly affect the performance of recursive algorithms that are not fast enough to follow the dynamics. For example, when we use an algorithm with slow adaptation and keep adaptation coefficients constant while significantly reducing the number of time points through downsampling, the estimates’ updates may no longer be sufficient to correctly reproduce variations in causal influences, resulting in a poor model of the investigated dynamics.

One limitation of our analyses is that we treated adaptation speed of the algorithm, model order and downsampling separately, while downsampling effects are likely related to the time scale of the interactions and selection of model order. Therefore, the choice of whether and how much to downsample not only depends on intrinsic characteristics of the recursive algorithm used, but also strongly depends on the temporal characteristics expected in the data. If temporal scales are unknown, a multiscale evaluation of directed interactions between processes may also be useful [68].

In the current study, we started by investigating three variables separately (adaptation coefficients, sampling rate and model order), because their effects on the intrinsic ability in connectivity estimation of the different recursive algorithms were still poorly understood. In the attempt to reduce the limitation mentioned above, we assessed the effects of downsampling in combination with the choice of model order by using a simulation framework similar to Simulation 3, but varying model order at each sampling rate (see S5 Appendix). The results of this simulation confirm our findings from Simulation 2 and Simulation 3 and do not contradict any of our previous conclusions. We further show that downsampling has an influence on the range of model orders that guarantee reliable and robust connectivity estimation. Overall, our supplementary results confirm that an excessive downsampling can have detrimental effects on the estimation performance of all recursive algorithms.

While we only considered recursive parametric methods for the estimation of connectivity measures, alternative tvMVAR methods also exist. For example, some algorithms are based on sliding window approaches, with the assumption of stationarity of signals in short time intervals [14,16]. The selection of window length is a critical issue for these approaches: on the one hand, a sufficient amount of data points is needed to accurately fit the MVAR model, but on the other, window length should be selected sufficiently small to guarantee the local stationarity of the data and to capture transient dynamic features.

Another class of approaches permits to approximate the spectral transfer function of the linear system and consequently estimating connectivity estimates, by using a two-steps process that consist of spectral estimation, usually through multitaper and wavelet transforms, followed by spectral factorization [11,69]. Such approaches are referred to as ‘nonparametric’ because they bypass the explicit estimation of MVAR models and the a priori selection of parameters such as model order and adaptation constant. However, even nonparametric approaches expect an initial choice of parameters, like for example taper function and number of tapers, mother wavelet and central frequency of wavelet transform, which may reduce spectral resolution. This limitation with respect to spectral resolution is not encountered in parametric methods [70]. A detailed comparisons and evaluation of non-parametric methods is an important topic for future work.

In both simulations and benchmark analyses, we used one connectivity measure [49] for all tvMVAR algorithms, because the purpose of this work was to provide a comparison between algorithms for tvMVAR modeling rather than between MVAR-based connectivity measures. In general, connectivity results and their interpretability may further depend on the choice of connectivity measure [71–73]. For example, strong differences between the amplitudes of the signals are known to affect the interpretability of PDC estimates, and thus revised definitions of PDC have been proposed to overcome or at least reduce the problem of scale variance [74–76]. Orthogonalized definitions of PDC have also been proposed to mitigate the effect of volume conduction and minimize the effect of mutual sources [41,59]. Spectral weighting of the PDC estimates can also improve the interpretability of the results [42,50]. It is worth to notice that since all variants of PDC are derived from a single estimation of the full multivariate model, they do not suffer from sensitivity issues due to model subset, which have been observed for the conditional Granger causality [77,78].

In summary, when model parameters are properly selected, the Kalman filter-based algorithms here considered can correctly model multivariate time series recorded from different brain areas and provide informative measures of the dynamic pattern of interactions between them. When temporal characteristics of the investigated neural process are not well known, the most reliable results can be expected from analyses based on single-trial modeling at relatively high sampling rates.

## Supporting information

S1 Appendix Preprocessing in benchmark EEG data. (DOCX)

S2 Appendix GLKF and the two strategies for multiple realizations. (DOCX)

S3 Appendix Computational instabilities using DEKF-AA in benchmark data. (DOCX)

S4 Appendix Effects of varying amount of trials. (DOCX)

S5 Appendix Downsampling and model order. (DOCX)
