# Supplementary material for: Time-varying MVAR algorithms for directed connectivity analysis: Critical comparison in simulations and benchmark EEG data
Source: PLoS One. 2018 Jun 11;13(6):e0198846. doi: 10.1371/journal.pone.0198846 (PMC5995381; doi:10.1371/journal.pone.0198846)
Supplement: S1 Appendix — (DOCX) [file pone.0198846.s001.docx]

**S1 Appendix: Preprocessing in benchmark EEG data**

In the benchmark dataset [1,2], we used a semi-automatic procedure based on two criteria to identify trials possibly contaminated with artifacts and guide the selection of trials to remove.

More specifically, trials were labeled as presumably bad when at least one of the following criteria was met:

1. Variance of signal in pre-stimulus interval [-100, 0 ms] exceeding variance in post-stimulus interval [0, 200 ms] for more than 3 channels.
2. Signal in pre-stimulus interval exceeding a threshold of 100 μV in at least one channel.

We performed the final selection of trials to remove by visual inspection. Number of remaining trials and list of excluded trials for each rat-stimulation condition are provided in S1 Table.

**S1 Table.**

**Results of the preprocessing procedures to remove bad trials.**

| **Rat-condition** | Number **of trials** | **Indices of removed trials** |
| --- | --- | --- |
| IC070523-STIMD | 32 | 3, 7, 10, 11, 16, 17, 23, 27, 29, 31, 32, 33, 35, 40, 41, 44, 45, 47 |
| IC070523-STIMG | 35 | 3, 10, 12, 13, 15, 16, 28, 29, 33, 35, 39, 42, 44, 48, 50 |
| RN060616A-STIMD | 45 | 14, 19, 21, 40, 49 |
| RN060616A-STIMG | 11 | 1, 2, 3, 4, 6, 8, 9, 11, 12, 14, 16, 18, 20, 21, 22, 23, 24, 25, 26, 28, 29, 30, 31, 32, 34, 35, 36, 37, 38 |
| RN060707A-STIMD | 18 | 1, 4, 5, 6, 9, 10, 12, 15, 16, 17, 19, 20, 21, 24, 25, 26, 28, 29, 30, 32, 33, 34, 35, 36, 38, 39, 44, 45, 46, 47, 49, 50 |
| RN060707A-STIMG | 16 | 1, 2, 5, 6, 8, 9, 10, 11, 12, 13, 15, 18, 20, 22, 23, 25, 27, 28, 29, 30, 31, 34, 35, 37, 38, 39, 42, 43, 45, 46, 47, 48, 49, 50 |
| RN060707B-STIMD | 43 | 6, 21, 27, 28, 29, 39, 40 |
| RN060707B-STIMG | 37 | 7, 10, 15, 16, 19, 24, 29, 32, 33, 34, 37, 41, 50 |
| RN060714C-STIMD | 38 | 3, 15, 16, 17, 20, 21, 23, 27, 30, 32, 44, 45 |
| RN060714C-STIMG | 37 | 1, 3, 8, 13, 14, 17, 18, 24, 32, 39, 42, 44, 46 |
| RN060728A-STIMD | 38 | 1, 2, 3, 4, 12, 14, 20, 21, 24, 30, 49, 50 |
| RN060728A-STIMG | 37 | 16, 17, 18, 21, 22, 25, 27, 30, 31, 38, 41, 48, 49 |
| RN060728B-STIMD | 27 | 2, 5, 7, 10, 11, 12, 13, 14, 16, 17, 22, 23, 24, 25, 28, 30, 31, 32, 34, 35, 39, 45, 46 |
| RN060728B-STIMG | 51 | 1, 2, 3, 4, 5, 12, 14, 16, 22, 23, 26, 27, 28, 30, 34, 35, 37, 39, 40, 41, 42, 43, 44, 46, 49, 51, 52, 53, 56, 57, 59, 60, 64, 65, 71, 72, 74, 77, 78, 81, 85, 89, 91, 92, 93, 97, 98, 99, 100 |
| RN060912-2-STIMD | 25 | 2, 7, 11, 12, 13, 14, 15, 17, 19, 20, 23, 25, 26, 27, 28, 29, 30, 31, 32, 36, 39, 40, 42, 44, 49 |
| RN060912-2-STIMG | 33 | 1, 7, 8, 10, 11, 14, 15, 16, 19, 21, 22, 24, 27, 28, 29, 44, 46 |
| RN060915A-2-STIMD | 34 | 7, 9, 10, 11, 14, 22, 27, 29, 30, 37, 40, 41, 42, 45, 46, 49 |
| RN060915A-2-STIMG | 40 | 6, 7, 13, 15, 18, 19, 27, 31, 41, 42 |
| RN070129C-2-STIMD | 28 | 1, 2, 4, 12, 18, 22, 23, 24, 26, 27, 28, 29, 32, 33, 34, 36, 41, 44, 46, 48, 49, 50 |
| RN070129C-2-STIMG | 22 | 1, 3, 6, 7, 8, 9, 13, 16, 17, 19, 21, 22, 23, 24, 25, 27, 29, 33, 34, 35, 36, 37, 40, 41, 42, 47, 48, 50 |

**References**

1. Plomp G, Quairiaux C, Michel CM, Astolfi L. The physiological plausibility of time-varying Granger-causal modeling: normalization and weighting by spectral power. NeuroImage. 2014;97: 206–216. doi:10.1016/j.neuroimage.2014.04.016

2. Quairiaux C, Mégevand P, Kiss JZ, Michel CM. Functional development of large-scale sensorimotor cortical networks in the brain. J Neurosci. 2011;31: 9574–9584. doi:10.1523/JNEUROSCI.5995-10.2011
