# Supplementary material for: Time-varying MVAR algorithms for directed connectivity analysis: Critical comparison in simulations and benchmark EEG data
Source: PLoS One. 2018 Jun 11;13(6):e0198846. doi: 10.1371/journal.pone.0198846 (PMC5995381; doi:10.1371/journal.pone.0198846)
Supplement: S2 Appendix — (DOCX) [file pone.0198846.s002.docx]

**S2 Appendix: GLKF and the two strategies for multiple realizations**

We here provide a proof of principle on the effect of multiple realizations on the estimation of GLKF, depending on the strategy used. We simulated time series using a VAR(2) system, in which node 1 and node 2 resonated at 40 Hz and 10 Hz, respectively. Signals in the two nodes were generated from independent white Gaussian noises with zero mean and unit variance. We used a sampling frequency of 120 Hz and generated 100 realizations of this system, each of length N=1800 (15 s). We imposed an interaction from node 1 to node 2 in the time interval between N/3 and 2N/3, by setting the corresponding autoregressive coefficient at lag 1 equal to 1, i.e., A_12_(1)=1; while, the interaction from node 1 to node 2 was zero in the first and the last thirds of each time series. We performed the estimation using single-trial modeling followed by averaging (GLKF-ST) and multi-trial modeling (GLKF-MT). In the time instants where a dynamic change was imposed to the interaction (N/3 and 2N/3), we computed the difference between the estimate at current time point and the estimate at the previous one. In this way, for each algorithm we measured the weight of estimate update at instant of transition, and then we compared this measure to the distribution of values obtained from performing the estimation on each single trial separately.

The results clearly show the difference between GLKF-ST and GLKF-MT (S1 Fig). The former allows obtaining smoother estimates with reduced variance, but there is no change in the adaptation speed compared to the estimation at the level of each single trial and the variations at instants of transition cannot be distinguished from the distribution obtained across 100 trials. A different behavior can be observed for GLKF-MT, where an increase in the amount of trials produces steeper variations in the recursive estimation and consequently makes the algorithm ‘intrinsically faster’, at the cost of higher variance in the estimates compared to GLKF-ST.


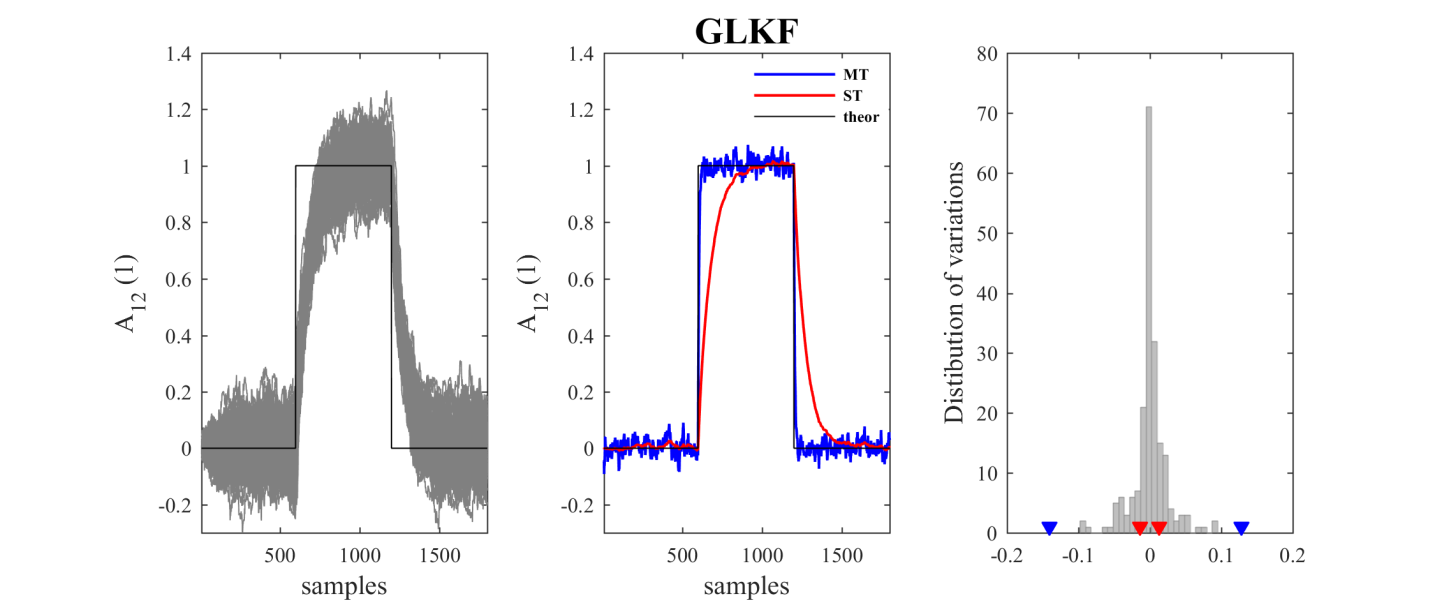


**S1 Fig. The two strategies for multiple realizations in GLKF.**

Left panel shows the imposed influence A_12_(1) (black) and the range of the estimates obtained from each single trial (gray). Central panel shows for A_12_(1) the theoretical values and the estimates obtained using GLKF-MT (blue) and GLKF-ST (red). Right panel shows the variations at the two points of transition for GLKF-MT (blue triangles) and GLKF-ST (red triangles), compared to the distribution of variations obtained across 100 trials (gray).
