# Supplementary material for: Time-varying MVAR algorithms for directed connectivity analysis: Critical comparison in simulations and benchmark EEG data
Source: PLoS One. 2018 Jun 11;13(6):e0198846. doi: 10.1371/journal.pone.0198846 (PMC5995381; doi:10.1371/journal.pone.0198846)
Supplement: S3 Appendix — (DOCX) [file pone.0198846.s003.docx]

**S3 Appendix: Computational instabilities using DEKF-AA in benchmark data**

In some conditions, using DEKF-AA we obtained invalid results, so called floating-point exceptions [1], due to computational instabilities. Under these “problematic” conditions, update matrices were ill-conditioned during the recursive estimation in DEKF-AA, i.e. we obtained for these matrices high condition numbers [2], which measure the sensitivity of the solution of a system of linear equations to errors in the data.

When AC=0.5 we obtained invalid results for 5 out of 10 animals. S2 Table provides the number of animals who showed invalid results with DEKF-AA varying model order (Fs=2000Hz): when *p* was above 10 we obtained invalid results in all the animals. Varying sampling rate, we obtained results in all datasets only at 500 Hz; while, invalid results were found for 9 out of 10 animals at original sampling rate (2000 Hz) and for 1 out of 10 after downsampling to 1000 Hz.

**S2 Table.**

**DEKF-AA: invalid results in benchmark EEG varying model order.**

| ***p*** |  | **2** | **4** | **6** | **8** | **10** | **12** | **14** | **16** |
| --- | --- | --- | --- | --- | --- | --- | --- | --- | --- |
| number of animals with invalid results |  | 0 | 4 | 4 | 9 | 10 | 10 | 10 | 10 |

**References**

1. Kahan W. IEEE standard 754 for binary floating-point arithmetic. Lect Notes Status IEEE. 1996;754: 11.

2. Belsley DA, Kuh E, Welsch RE. Regression Diagnostics: Identifying Influential Data and Sources of Collinearity [Internet]. Hoboken, NJ, USA: John Wiley & Sons, Inc.; 1980. doi:10.1002/0471725153
