# Supplementary material for: Time-varying MVAR algorithms for directed connectivity analysis: Critical comparison in simulations and benchmark EEG data
Source: PLoS One. 2018 Jun 11;13(6):e0198846. doi: 10.1371/journal.pone.0198846 (PMC5995381; doi:10.1371/journal.pone.0198846)
Supplement: S4 Appendix — (DOCX) [file pone.0198846.s004.docx]

**S4 Appendix: Effects of varying amount of trials**

We here assessed how the number of trials affects estimation performance of the six recursive algorithms. We used the 5-nodes surrogate network from Simulation 1 while varying the number of simulated trials (*k* = 1, 2, 5, 10, 20, 40). All other details were as in Simulation 1.

The results showed generally constant model quality across levels of trials when the number of trials exceeded 5 (S2 Fig–panels A-B). In most algorithms we obtained slightly increased GOF and percent consistency when using 1 or 2 trials only, which may reflect overfitting to the observed data. Differently, for GLKF-MT the percent consistency was lower using 1-2 trials with respect to higher levels of trials.

In terms of connectivity estimation, compared to the other algorithms GLKF-MT showed a steeper initial reduction in misses with increasing number of trials (S2 Fig–panel C), and an overall stronger positive effect of trials in improving the estimation of simulated connections. The positive effect of increasing number of trials was also present in the other algorithms, but showed no further improvements beyond 10 trials. False alarms showed similar behavior across all algorithms (S2 Fig–panel D), with slightly higher benefit by increasing number of trials for GLKF-ST and MVAAR-ST.

Increasing number of trials also improved the ability of GLKF-MT in correctly detecting peak latencies of driving, while results were basically constant across trials for the remaining algorithms (S2 Fig–panel E), but characterized by reduced CIs increasing trials.

In sum, our findings confirm that increasing the number of trials is beneficial for recursive algorithms. However, there is a certain amount of trials after which the measures of estimation accuracy show a plateau, suggesting that there is only little benefit in adding more trials beyond that amount.


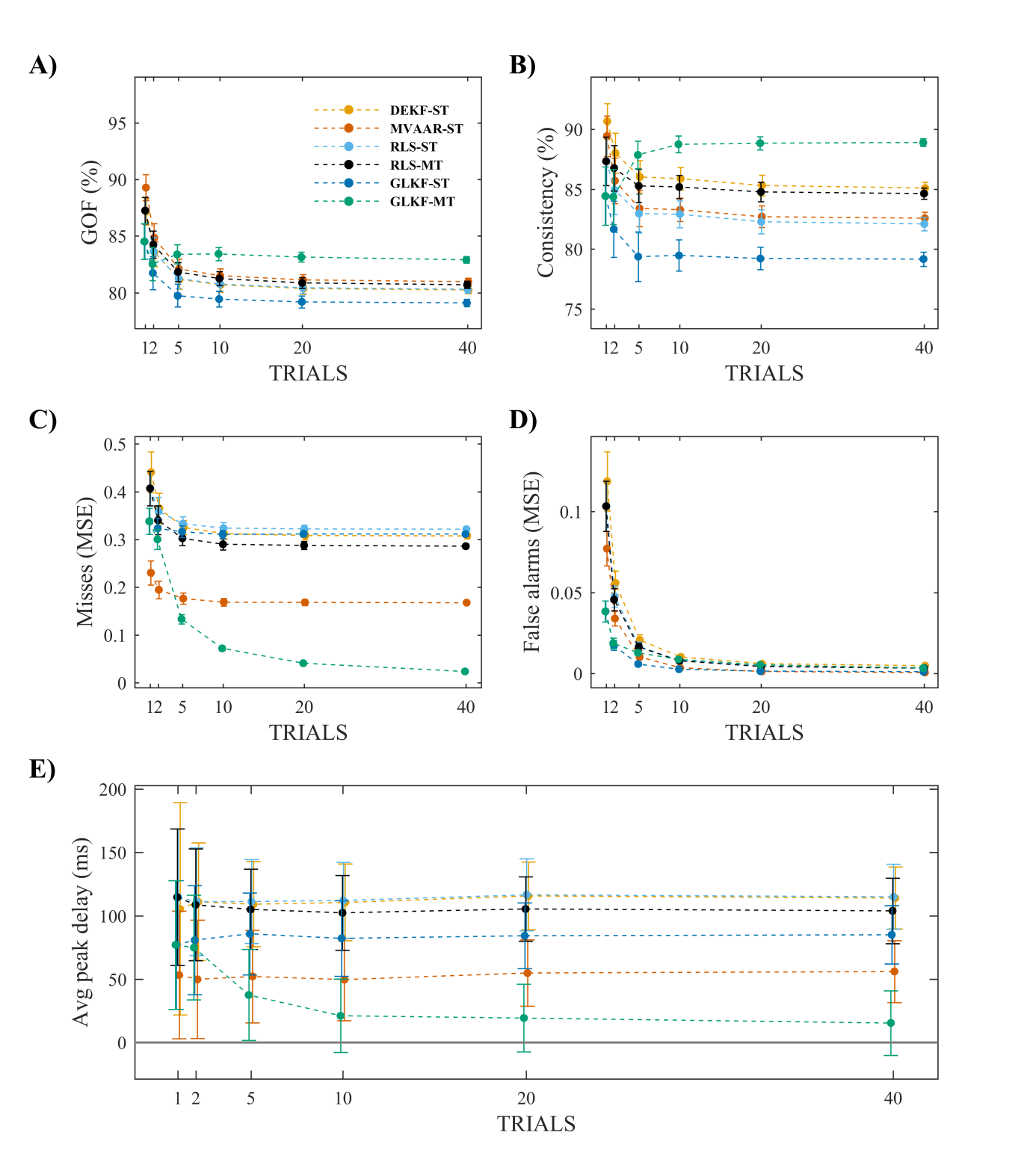


**S2 Fig. Simulation 4 on the effects of varying amount of trials.**

A) Shows the goodness-of-fit (GOF). B) Shows the percent consistency. C) Shows the misses (normalized MSE), which are computed on the edges with simulated connections (Fig 1A). D) Shows the false alarms (normalized MSE), computed on the edges with null connections. E) Shows the average peak delay across the four imposed connections in the 5-nodes model (Fig 1B). The results in each plot are shown as a function of total amount of trials, for the four recursive algorithms and the two ways of exploiting multiple trials: single-trial modeling (ST) and multi-trial modeling (MT), available only for RLS and GLKF. Error bars represent 95% CI of the mean value computed across 50 simulations.
