# Supplementary material for: Time-varying MVAR algorithms for directed connectivity analysis: Critical comparison in simulations and benchmark EEG data
Source: PLoS One. 2018 Jun 11;13(6):e0198846. doi: 10.1371/journal.pone.0198846 (PMC5995381; doi:10.1371/journal.pone.0198846)
Supplement: S5 Appendix — (DOCX) [file pone.0198846.s005.docx]

**S5 Appendix: Downsampling and model order**

To investigate whether the effects of downsampling depend on the model order choice we used a variation on Simulation 3 in which we performed the analyses varying model order (1 to 20 in steps of 1) and sampling rate (1000 Hz to 100 Hz in steps of 100 Hz) in fully-crossed way. The duration of the causal influence from node 1 to node 2 was 150 ms and time-frequency connectivity analysis was performed up to 50 Hz. We repeated the generation-estimation procedure 50 times for each of the fully-crossed conditions considered.

All six algorithms showed generally high GOF and uniform across model orders at each level of sampling rate down to 400-300 Hz (S3 Fig–A); while downsampling to 200 Hz and 100 Hz resulted in stronger dependence on model order and overall detrimental effect on GOF. We observed similar results also for the percent consistency (S3 Fig–B), except with MVAAR-ST and GLKF-ST, which showed lower percent consistency (as in Simulation 2). An excessive downsampling produced a remarkable decrease in performance also in this case.

Results in terms of connectivity estimation confirmed the previous findings of our study. More specifically, the results for misses reproduced a specific behavior for all algorithms at each sampling rate (S4 Fig–A). Such behavior was characterized by (i) minimum MSE in misses for a model order close to the value that matches the imposed lag, (ii) increased MSE for model orders smaller than the lag, and (iii) quite stable MSE for a certain range of model orders exceeding the lag. This latter characteristic (iii) was also dependent on the downsampling. For most of the algorithms, by increasing model order beyond the optimal value we observed an increase in misses, which was almost flat at original sampling rate and became steeper at lower sampling rates; on the other hand, GLKF-MT and RLS-MT showed a more robust behavior varying model order in the range beyond the optimal value, even after downsampling. Moreover, the results confirmed that for all algorithms downsampling can decrease false alarms and that increasing model order produces a linear increase in false alarms (S4 Fig–B). GLKF-MT showed the strongest dependence on downsampling and model order choice for false alarms.

In terms of temporal discrimination, the majority of algorithms produced a systematic overestimation of peak latency across sampling rates and model orders (S4 Fig–C), characterized by increased delays at lowest sampling rates, in line with the results of Simulation 2 and Simulation 3. Overall, GLKF-MT guaranteed the best temporal discrimination of peak latency for values of model order in the range around the one matching the imposed lag at each sampling frequency. As previously shown in Simulation 2, high variability in the results across simulations was observed in GLKF-MT, but only at original sampling rate and when the model order was 17 or bigger.

In sum, we considered variations in sampling rate and model order in a fully-crossed way, and the results did not contradict the previous findings from Simulation 2 and Simulation 3. The results of this simulation support the finding that the best estimation of a simulated connection can be generally obtained using a range of model orders around the one that matches the imposed lag of the connection itself at each sampling rate. We further show that the effects of downsampling are qualitatively similar across model orders and that they generally depend on the characteristics of the considered algorithm. For example, we confirmed a beneficial effect of downsampling on connectivity estimation for GLKF-MT, due to a substantial reduction in false alarms, while showing robustness in terms of misses under the conditions here considered. Overall, an excessive downsampling (200-100 Hz) produced detrimental effects on the performance of all algorithms, both in terms of model fitting and connectivity estimation.


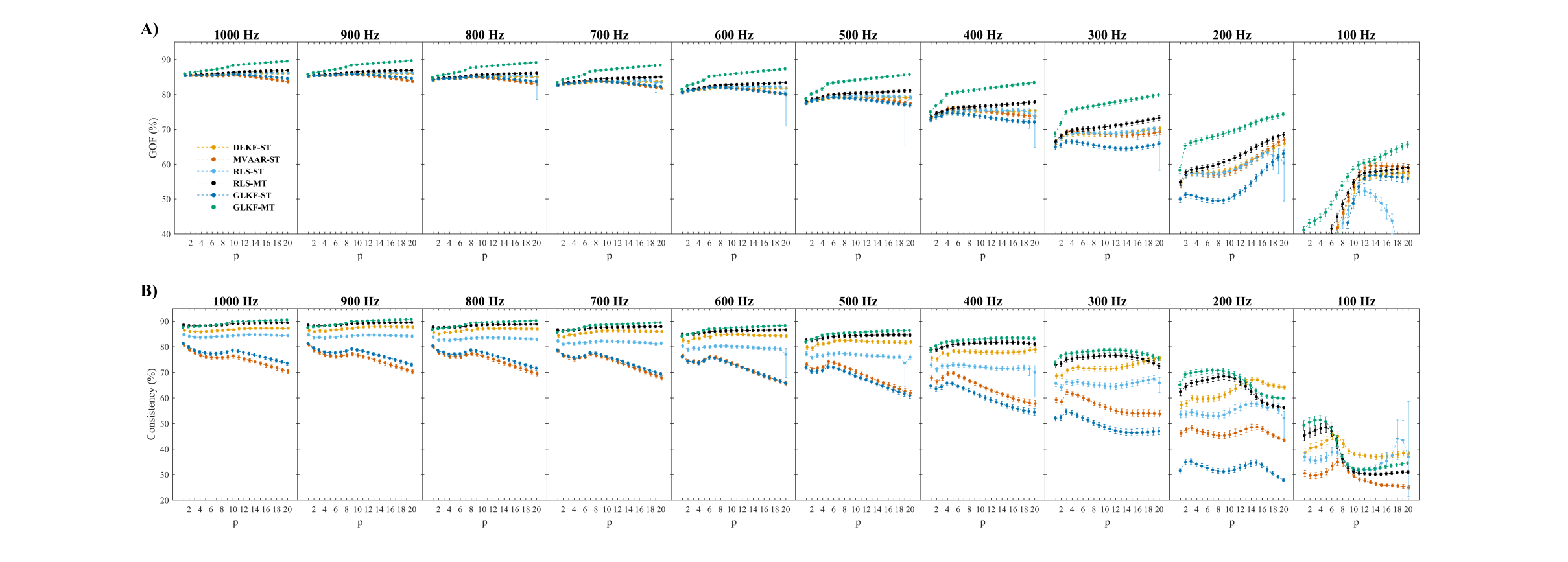


**S3 Fig. Simulation 5 on the effects of varying sampling rate in combination with model order: model fitting.**

The pictures show the results in terms of the two model fitting measures: A) goodness-of-fit (GOF); B) percent consistency. The results are shown from left to right for sampling rates from 1000 Hz to 100 Hz in steps of 100 Hz. In each plot the results are reported varying model order *p*, for the four recursive algorithms and the two ways of exploiting multiple trials: single-trial modeling and multi-trial modeling, available only for RLS and GLKF. Error bars represent 95% CI of the mean value computed across 50 simulations.


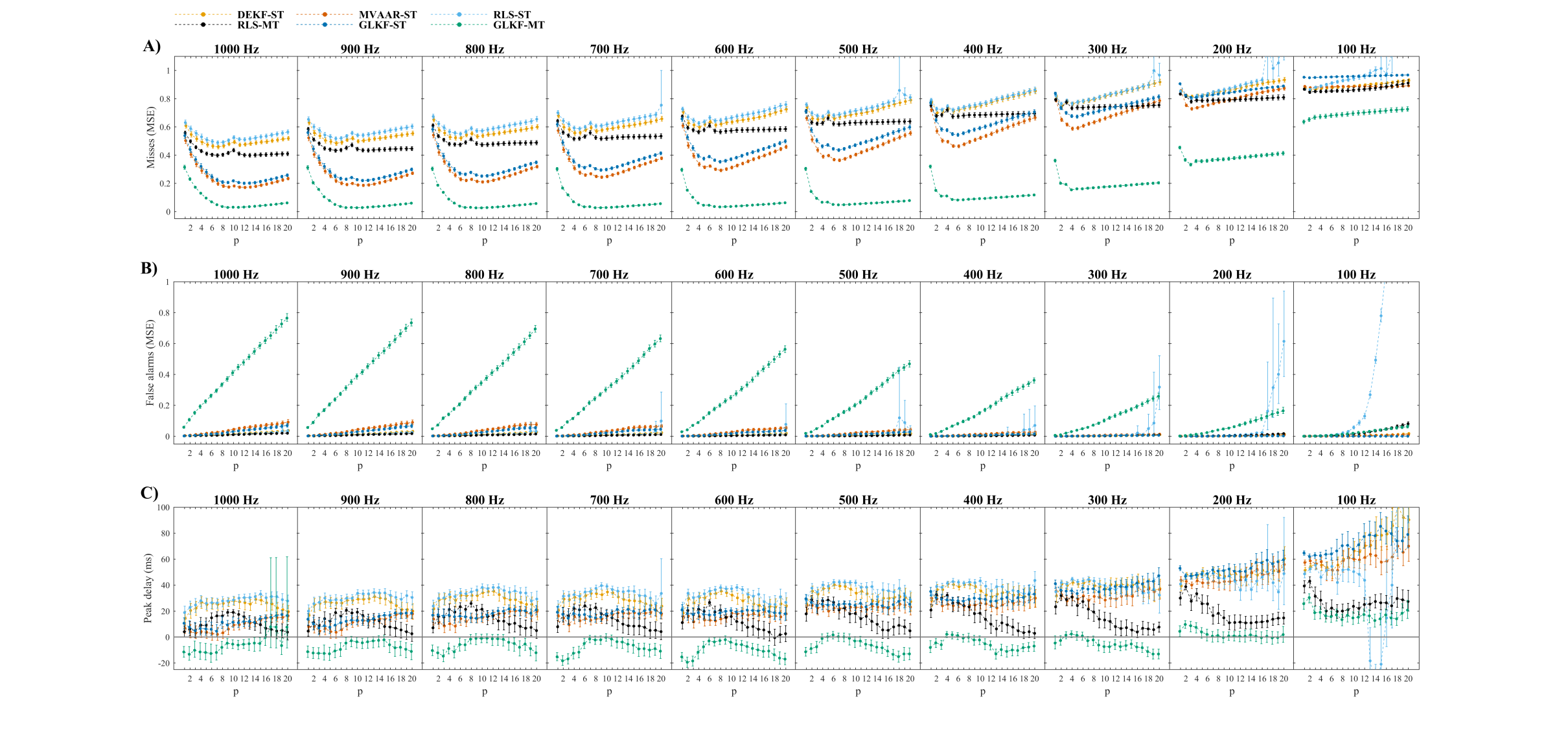


**S4 Fig. Simulation 5 on the effects of varying sampling rate in combination with model order: connectivity estimation.**

A) Shows the misses, which are computed as normalized mean squared differences between estimated connectivity values and those imposed from node 1 to node 2 (Fig 1C). B) Shows the false alarms, which are computed as normalized mean squared error on the null connection from node 2 to node 1 (Fig 1C). C) Shows the peak delay, which is computed as difference between peak latencies of true and estimated causal influence from node 1 to node 2 (Fig 1C). The results are shown from left to right for sampling rates from 1000 Hz to 100 Hz in steps of 100 Hz. In each plot the results are reported varying model order *p*, for the four recursive algorithms and the two ways of exploiting multiple trials: single-trial modeling and multi-trial modeling, available only for RLS and GLKF. Error bars represent 95% CI of the mean value computed across 50 simulations.
